# Synergistic charge-transfer dynamics of novel pyridoquinazolindone-containing triphenylamine-based push–pull chromophores: from structural optimization to performance metrics in photovoltaic solar cells and static, dynamic, solvent-dependent nonlinear optical response applications†

**DOI:** 10.1039/d4ra05290k

**Published:** 2024-10-14

**Authors:** Sehar Nadeem, Abida Anwar, Muhammad Usman Khan, Abrar Ul Hassan, Khalid Abdullah Alrashidi

**Affiliations:** a Department of Chemistry, University of Okara Okara 56300 Pakistan usman.chemistry@gmail.com usmankhan@uo.edu.pk; b Lunan Research Institute, Beijing Institute of Technology 888 Zhengtai Road Tengzhou 277599 China; c Department of Chemistry, College of Science, King Saud University Riyadh 11451 Saudi Arabia

## Abstract

Modern technological breakthroughs depend on nonlinear optical (NLO) and photovoltaic (PV) materials, essential for creating advanced photonic devices and efficient solar cells. Herein, the NLO, PV, electrical, and photophysical characteristics of proposed chromophores (WLK-1–WLK-6) designed from pyridoquinazolindone-containing triphenylamine have been systematically altered by the addition of different spacers categorized as K1, K2, K3, K4, K5, and K6 (named as i-series). This fine-tuning was accomplished using TD-DFT, DFT computations, and the Scharber model. The impact of spectrum of medium polarity, ranging from the least polar to the most polar, including water (*ε* = 78.36), methanol (*ε* = 32.61), DMSO (*ε* = 46.83), tetrahydrofuran (*ε* = 7.43), benzene (*ε* = 2.27) and chloroform (*ε* = 4.71), is explored in detail utilizing the IEFPCM model on NLO and PV properties. Moreover, the response of different analyses like DOS, NCI, NBO, FMO, dipole moments (*µ*), and hyperpolarizability (*β*) in both gas, polar and non-polar solvents was analyzed. Our structure–property relationship studies revealed that adding extra spacer groups, particularly those containing thiophene spacers, considerably impacted the lowering of the energy gap (3.853–4.190 eV). The simulated UV-Vis spectra illustrate significant π → π* transitions and lower n → π* transitions, primarily in the near-infrared (IR) range of 558.613 to 429.844 nm. Push–pull chromophores showed extraordinary frequency-dependent NLO properties, SHG *β*(−2*ω*, *ω*, *ω*), and EOPE *β*(−*ω*, *ω*, 0) effect computed at laser frequencies of 1064 and 532 nm. Among the proposed compounds, WLK-6 with the K6 spacer demonstrated a smaller energy gap (3.853 eV), resulting in a maximum optical absorption peak at *λ*_max_ = 558.613 nm and the maximum hyperpolarizability in benzene (9.00 × 10^4^ a.u.), methanol (1.22 × 10^5^ a.u.), THF (1.12 × 10^5^ a.u.), DMSO (1.23 × 10^5^ a.u.), and water (1.23 × 10^5^ a.u.). Our study found that WLK-6, WLK-5, and WLK-1 compounds also had good photovoltaic (PV) capabilities, reaching a power conversion efficiency (PCE) of around 5% and an injection efficiency (Δ*G*^inject^) of 0.191. In addition to these analyses, we performed topologic studies, such as TDM, ELF, NCI, MEP, LOL, and electron–hole overlap plots to better understand both intra and intermolecular interactions. Based on these results, it is clear that modifying longer π-linker groups in A–D–π–A conjugated systems benefits the optoelectronic characteristics and NLO responses for organic PV devices.

## Abstract 2

Modern technological breakthroughs depend on nonlinear optical (NLO) and photovoltaic (PV) materials, essential for creating advanced photonic devices and efficient solar cells.

## Graphical abstract

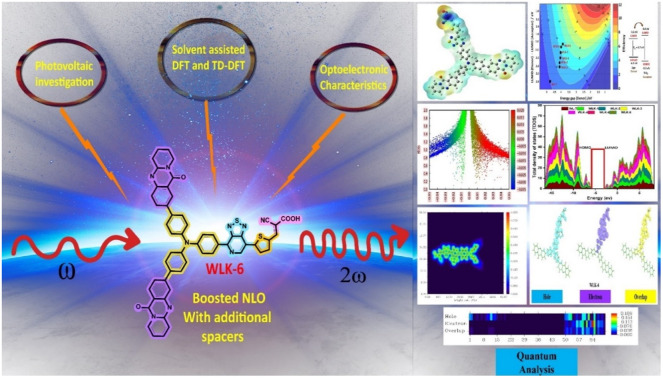

## 1. Introduction

In today's high-tech world, non-linear optical (NLO) materials are regarded as the most advanced compounds because they alter laser light's frequency and phase.^1^ Research on nonlinear optical (NLO) materials is a dynamic field involving theoretical and experimental scientists.^2–5^ This field is driven by the wide range of applications in photonics, nanophotonics, optoelectronics, and optics.^6–10^ Many NLO materials, including polymers, inorganic semiconductors, organic semiconductors, nanomaterials, and molecular dyes, are being studied in great detail in present-day studies.^11–15^ Metal-free organic materials, due to low dielectric constants, flexibility, low production costs, high photoelectric coefficients, ease of modification, and versatility in manufacturing and customization, are gaining popularity right now. These distinguishing characteristics make them superior candidates for nonlinear optical (NLO) applications compared to other materials. Intramolecular charge transfer, conjugation length, and conformation of molecules are some of the factors that influence the NLO characteristics.^8,11,12^ To enhance the performance of NLO, a variety of strategies have been adopted, including the designing of molecules with different donors, conjugated π-linkers, and acceptor moiety.^13–15^ It has been proven that the Acceptor–Donor–π–Acceptor (A–D–π–A) molecules exhibit excellent electron acceptor properties in organic photovoltaics (OPV), resulting in high efficiency of power conversion (PCE) in OPV.^16–18^ Furthermore, A–D–π–A configuration molecules are anticipated to exhibit a significant NLO characteristic, attracting much interest from modern researchers because of intramolecular charge transfer (ICT), highly conjugated π-systems, and high electron mobility. In addition, organic dyes' ease of synthesis, cost-effectiveness, and environmental friendliness has earned a lot of attention.^19–21^ Although there are many different donor–acceptor-based D–A–D, D–A, D–A–D–A–D–A, and D–A–A push–pull model configurations documented in the previous literature,^8,13,22^ A–D–π–A type structures are the most frequently studied ones.^23,24^ It is possible to fine-tune NLO properties of A–D–π–A based compounds by adding additional acceptor and donor moieties, as well as by substituting them.^25,26^ Although building blocks (donor, acceptor, and π-spacer) are essential for attaining amplified NLO responsiveness, the addition of more spacers is also considered important. Furthermore, a variety of π-linkers described in the literature improve electron density transmission towards the donor acceptor. A–D–π–A organic molecules combine suitable D, π-bridge, and A units to produce a push–pull architecture. These push–pull designs improve asymmetric electronic distribution, minimize charge recombination, induce charge separation, widen the absorption range to wider wavelengths, lower the energy gap, and boost the NLO response.^27–29^ The literature is brimful with A–D–π–A compounds including electron-rich phenothiazine, carbazole, indoline, coumarin, porphyrin, fluorene, and triarylamine as donors (D), heterocyclic compounds as π-linkers, and electron-deficient carboxylic acids as acceptors (A) for diverse applications. These A–D–π–A compounds have been thoroughly researched for their DSSC characteristics.^16,30,31^ however, there is a lack of significant literature regarding their investigation into NLO characteristics. Consequently, it is crucial to focus on designing and investigating these chromophores that exhibit efficient NLO features. In line with our continuous interest in creating and advancing these NLO substances in addition to their use as sensitizers,^32^ we present WL-7, a dye synthesized by Yang Gao *et al.*,^33^ from the literature. Based on the existing literature and our understanding, no comprehensive theoretical investigation has been conducted into the NLO characteristics or first hyperpolarizability of WL-7. We plan to conduct an initial screening and development of this A–D–π–A organic chromophore using first-principles methods to explore its possible NLO response properties. WL-7 having A–D–π–A configuration is generated using a simple synthetic route presented in Scheme S1 (ESI).† Moreover, WL-7 is a metal-free organic dye based on triphenylamine, designed with a pyridoquinazolinone-containing triphenylamine core, where triphenylamine serves as the electron donor, cyanoacrylic acid as the electron terminal acceptor, thiophene unit as the π-conjugated spacer, pyridoquinazolinone as the auxiliary acceptor. In this study, we used WL-7 as a prototype and designed a series of unique organic chromophores called WLK-1 to WLK-6 by inserting a second π-bridge beside the first π-bridge of WL-7.

TD-DFT and DFT were used to calculate the electronic features of designed compounds through charge analysis, population analysis, NBO analysis, and optical and non-linear optical responsiveness (*β*_tot_) of WL-7 and WLK-1–WLK-6. This quantum chemistry study intends to show how different π-bridges affect the possible NLO features of the created molecules. We also evaluated these developed chemicals as DSSC's photosensitizers. It is inferred that these compounds will greatly enhance the NLO performance of A–D–π–A compounds in modern optical and photovoltaic systems.

## 2. Quantum methodology

**Fig. 1:**
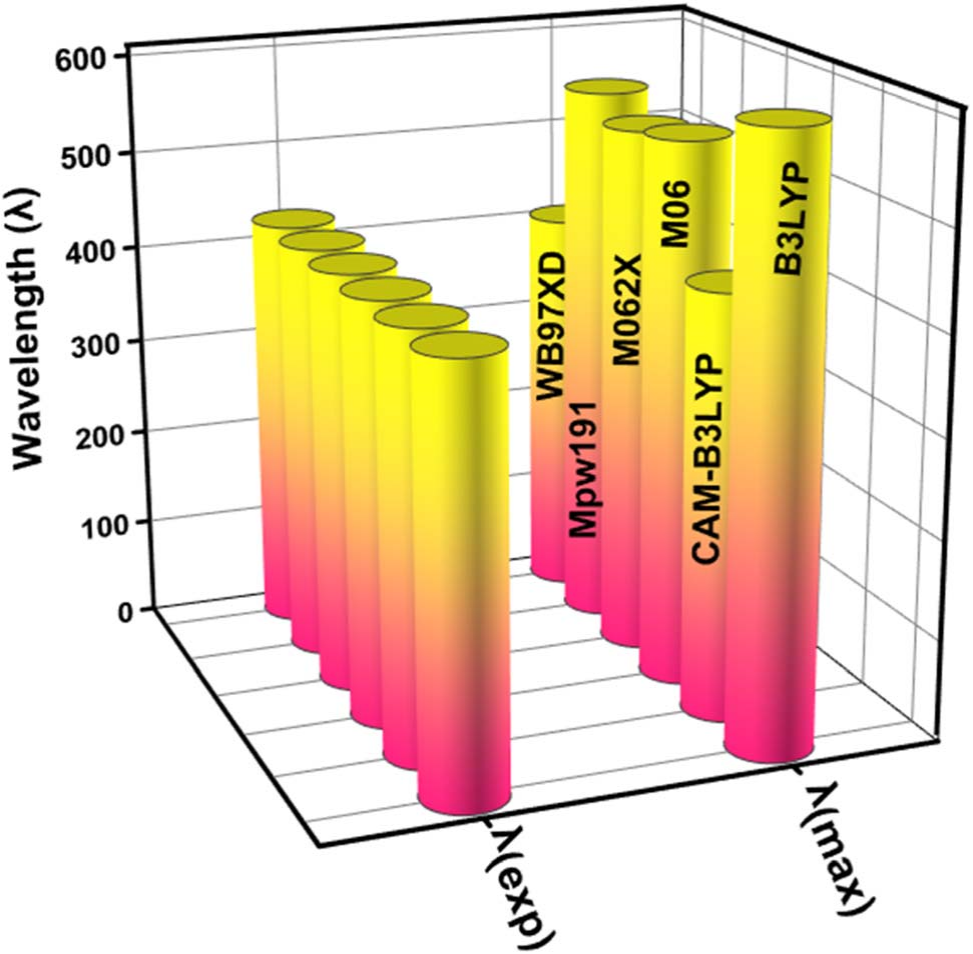
The graphical representation for comparing the theoretical (*λ*_max_) with experimental absorption wavelength (*λ*_exp_) by applying different functionals (XC).

This study used the Gaussian 09 software package,^34^ for all quantum chemical simulations. GaussView 5.0 (ref. 35) was used to generate the input files. In the gas phase, the 6-31 G(d,p) basis set was combined with five (XC) functionals (M062X, CAM-B3LYP, B3LYP, WB97XD, and MPW1PW91) to optimise the reference compound (WL-7) without symmetry constraints. The maximum absorption spectrum (*λ*_max_) of WL-7 was calculated using TDDFT and the aforementioned functionals. The findings show that, when utilizing the XC functionals, the vertical excitation energies of the WL-7 prototype were overestimated by 177 nm, 2 nm, 125 nm, 115 nm, 134 nm, and −22 nm compared to the experimental value of 428 nm.^33^ Thus, the CAM-B3LYP functional was chosen based on a 2 nm difference. So, the concordance between calculated and reported experimental results using the CAM-B3LYP/6-31G(d,p) functional suggests employing this functional and basis set combination for further calculations in the current study, as represented in Fig. 1. After optimising the molecules under investigation, the CAM-B3LYP/6-31G(d,p) functional was used for a vibrational study. This validated the absence of negative Eigen values and the presence of optimised forms in potential energy surfaces at their real minimum. The UV-Vis spectra were calculated using the conductor-like polarisable continuum model (CPCM)^36,37^ and a methanol solvent at the aforementioned basis set and theory. Moreover, the parameters of the FMOs-especially the HOMO–LUMO energy gap (*E*_g_), the lowest unoccupied molecular orbital (LUMO), and the highest occupied molecular orbital (HOMO) were then obtained using the optimized molecular geometry. The TDOS spectrum was calculated, and simulated absorption spectra of UV-vis were generated using TD-DFT. Hence, recent improvements have enabled the precise prediction of electrochemical behaviours as well as the calculation of photophysical properties such as UV-vis absorption (calculated experimentally using cyclic voltammetry (CV) measurements) through the application of DFT and its time dependent TD-DFT.^38–40^ We employed a this methodology to explore the characteristics of the materials under examination, employing a range of techniques including localized-orbital locator (LOL), reduced electron density gradient (RDG) plots, electron localization function (ELF) and transition density matrix (TDM) for a comprehensive evaluation of non-covalent interactions and charge transfer capabilities. The investigation was conducted utilizing the Multiwfn 3.7 software program,^41^ and the contour plots were seen using the VMD (Visual Molecular Dynamics) program.^42^ Photovoltaic parameters were estimated, and the power conversion efficiency (PCE) was ascertained using the Scharber diagram.^43^ The electric dipole moment, polarizability, and first hyperpolarizability were all computed at an aforementioned DFT level to examine the nonlinear optical characteristics.

### 2.1 Selection of model for solvent-dependant NLO calculation

Along with the above methodology, solvent-dependent non-linear optical characteristics are also estimated. These computations were performed using IEF-PCM (the integral equation formalism polarized continuum model) in a variety of solvents, including water (H_2_O), methanol (CH_3_OH), dimethyl sulfoxide (DMSO), and tetrahydrofuran (THF).^44^ The Integral Equation Formalism Polarisable Continuum Model (IEFPCM) was most likely used to calculate the NLO characteristics of solvents such as water, benzene, and ethanol for several reasons: IEFPCM provides a more realistic description of the solute–solvent interface than simpler models such as the Onsager model.^45^ It employs a molecule-shaped cavity and the complete molecular electrostatic potential, resulting in a more accurate simulation of the solvent environment. This is critical for determining features like NLO coefficients sensitive to electronic structure. IEFPCM is computationally efficient and scales well across system sizes. It is frequently faster than more comprehensive explicit solvent models (necessitating modelling individual solvent molecules) while maintaining a good approximation of solvent effects. Many computational chemistry software packages have IEFPCM support with default parameters suitable for various solvents. Furthermore, IEFPCM has been frequently used in the literature, so its merits and weaknesses are well-documented.^46,47^ Because of its widespread use, IEFPCM results have been validated against experimental data, giving researchers confidence in the model's performance for similar systems.

Moreover, the current work also shows how the properties of developed molecules' laser-dependent NLO are assessed.

## 3. Results and discussion

### 3.1 Structural configuration

**Fig. 2:**
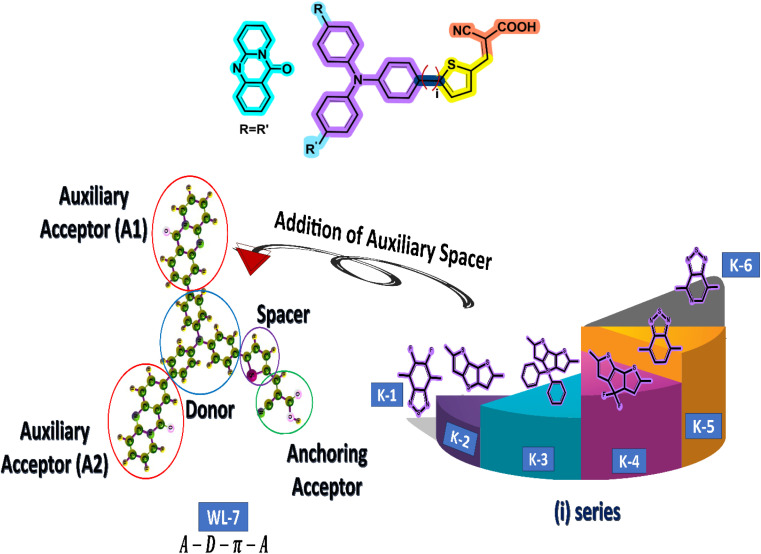
The schematic representation for designing different chromophores. First, the WL-7 is referenced with two auxiliary acceptors A1 and A2. Then, we have added different π-spacers (named i-series) with thiophene.

The current study employs quantum chemical methods to elucidate the theoretical design and investigation of a new organic push–pull chromophore. To develop exceptional nonlinear optical (NLO) compounds, the experimentally prepared dye WL-7 (depicted in Fig. 2) is utilized. The structure of WL-7 comprises four segments: Triphenylamine acting as the donor moiety, thiophene serving as the π-linker group, and 2-cyanoacrylic acid as the acceptor units along with two auxiliary acceptor substitutions (R=R′) identified as 11*H*-pyrido[2,1-*b*]quinazolin-11-one attached to the triphenylamine donor. Following this modification, the structure transforms into an A–D–π–A configuration. As previously stated, the π-spacer and acceptor components significantly influence the modulation of intramolecular charge transfer (ICT) properties, absorption characteristics, and the HOMO–LUMO energy gap. Consequently, we designed a series of push–pull compounds, WLK-1 to WLK-6, to create effective NLO candidates. We achieved this by using the WL-7 as a prototype and modifying the π-spacer block with various π-spacers we chose from the literature.^48^ While the thiophene spacers are modulated with additional spacers named K-1 to K-6 (as illustrated in Fig. 2), which have preferred planarity and electron-richness than thiophene, the donor and acceptor blocks are kept unaltered throughout the designing process.

**Fig. 3:**
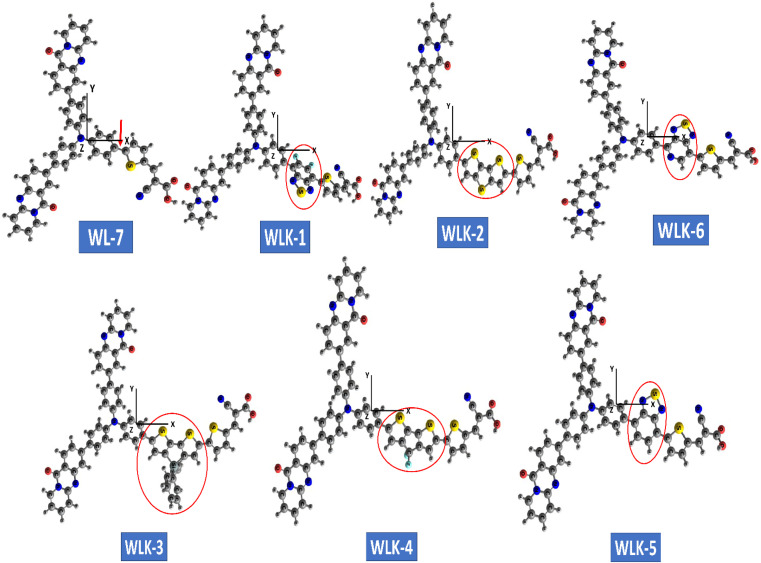
The ball and stick configuration of optimized push–pull chromophores viewed through GaussView with atom labels.

The optimized configuration of all investigated molecules (WL-7, WLK-1, and WLK-6) had been computed at the CAM-B3LYP/6-311G(d,p) levels. Fig. 3 shows the optimized structures of the proposed strategy. Fig. S1 (ESI)† displays the 2D sketch mapping for all developed molecules. To provide specific directions for building novel NLO compounds and to clarify the effects of distinct π-spacers on the photophysical, electrical, and NLO response properties, DFT and TD-DFT simulations were performed on WL-7 and WLK-1 to WLK-6. Within this framework, the following fundamental characteristics are as follows: (1) electronic characteristics; (2) polarizability (*α*); (3) NBO investigation; (4) hyperpolarizability (*β*); (5) photovoltaic features; and (6) absorption properties.

### 3.2 Charge analysis

**Fig. 4:**
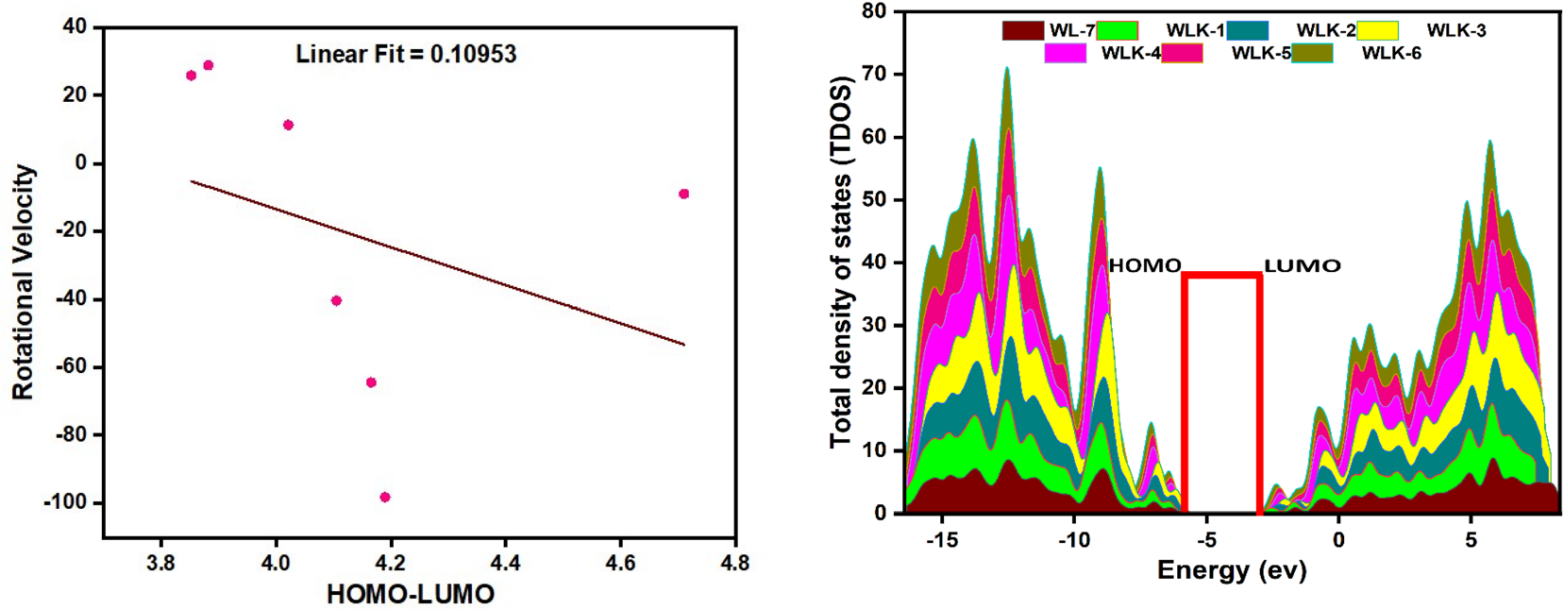
The graphical representation of the correlational analysis of rotational velocity and HOMO–LUMO energies (left-side) and TDOS showing population analysis (right-side).

FMO analysis is a valuable method for assessing materials' charge distribution and electronic configuration. It is notably beneficial in studying photovoltaic (PV) and nonlinear optical properties because it provides extensive information about a material's electronic structure, energy levels, and charge transfer mechanisms.^49,50^ FMO study of WL-7 and WLK-1–WLK-6 revealed that the donor moiety has charge densities of LUMO and HOMO. This discovery emphasizes the importance of the donor component in determining the electrical properties and characteristics of these materials in PV and nonlinear optical applications. On the other hand, some charge presence was found on the acceptor fragment and a marginally greater electronic charge was noted on the π-bridge moiety. This distribution indicates that these chromophores' electronic configuration and collective NLO response arise from the participation of all fragments, specifically the donor, spacer groups, and anchoring acceptor. The results of the FMO study for WL-7 and WLK-1 to WLK-6 showed that the charge of LUMO was not solely present on the auxiliary acceptor but rather was distributed throughout the entire skeleton of the proposed compounds. This arrangement indicates a clear charge separation between the acceptor and donor components, potentially enhancing the NLO properties of the chromophore. Rotational energy affects the symmetry and general molecular structure, affecting the NLO characteristics. This energy can modify the arrangement and positioning of molecules, leading to changes in their nonlinear optical properties. Adjusting the rotational energy allows one to influence a material's NLO response. The correlations between rotational energy and HOMO–LUMO values reveal information on the connection among molecular structure, electronic transitions, and NLO responses (see Fig. 4). By examining these relationships, it is possible to reveal patterns and trends that assist in developing and enhancing push–pull chromophores.^51^ From Table S1 (ESI),† it was observed that HOMO–LUMO energy trend is WLK > WLK-2 > WLK-3 > WLK-4 > WLK-5 > WLK-1 > WLK-6. And rotational velocity shows following results: WLK-1 > WLK-6 > WLK-5 > WLK > WLK-4 > WLK-3 > WLK-2. From these trend it was demonstrated that elevation in rotational velocity was observed not to influence HOMO–LUMO. Moreover, the slope in Fig. 4, represents the rate of change between the dependent variable (*y*) and the independent variable (*x*). And, a small linear fit value indicates a weak relationship between the rotational velocity and HOMO–LUMO energy gap. Hence, it was predicted that with the increase or decrease in rotational velocity, there is no effect on energy gap.

**Fig. 5:**
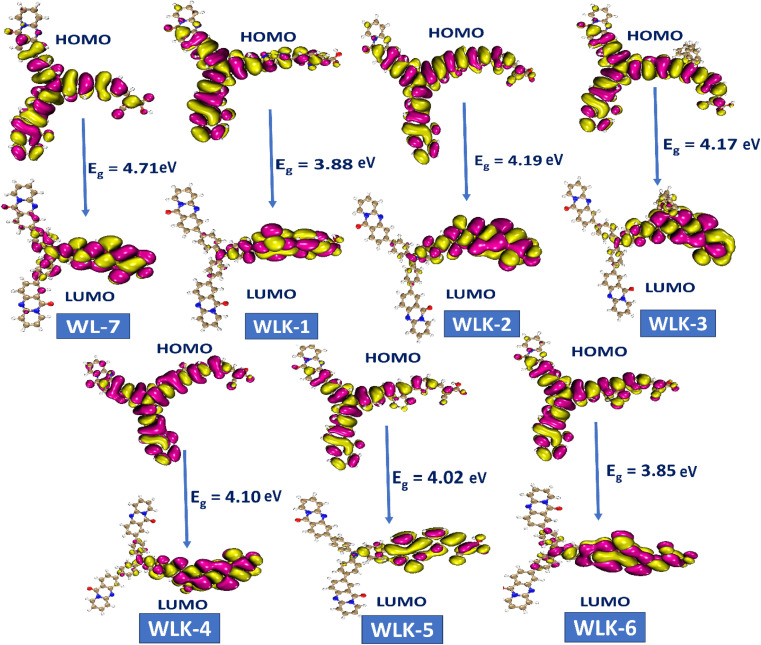
The contour surface represented the HOMO–LUMO and energy gap values for all designed compounds.

The study on FMO indicated that positive and negative charges in molecular orbitals were delocalized. Moreover, atoms participating in π-bonding, conjugated systems, and electronegative atoms enhanced delocalization, as illustrated in Fig. 5. These atoms are crucial for spreading electron density and stabilizing the molecule. The creation of π-bonds through overlapping π-orbitals allows for charge delocalization.^52^ electronegative atoms can decrease electron density through inductive or resonance mechanisms, thus aiding in charge redistribution. These particular atoms promote enhanced charge dispersion within the molecular structure. Additionally, the HOMO and LUMO energy levels, along with the intramolecular charge transfer (ICT) from donor (D) to acceptor (A) *via* two π-linkers, play vital roles in the characteristics of these compounds. The electron density distributions of FMOs depicted in Fig. 5 serve to illustrate the charge transfer phenomenon.

Molecular systems with lower energy gap (*E*_gap_) values exhibit high polarizability and significant intramolecular charge transfer (ICT) from the pendant donor group (D) to the acceptor unit (A) through a π-conjugated bridge. This transfer from D to A enhances the absorption range towards longer wavelengths and the nonlinear optical (NLO) response.^53,54^ Consequently, the *E*_HOMO_, *E*_LUMO_, and *E*_gap_ values of WL-7 and WLK-1 to WLK-6 were calculated, and the outcomes are displayed in Table S1 (ESI).† Table S1† shows that WL-7 has computed *E*_HOMO_ and *E*_LUMO_ energy levels of −6.349 and −1.639 eV, respectively, and an *E*_gap_ value of 4.710 eV, making it the compound with the highest *E*_gap_ value among those tested. However, in the designed compounds, the energy gap begins to narrow. WLK-2 has an *E*_gap_ value of 4.190 eV, which decreases to 4.166 eV in WLK-3, 4.105 eV in WLK-4, and 4.021 eV in WLK-5. WLK-1 has a lower band gap, with an *E*_gap_ value of 3.882 eV, which can be attributed to the additional spacer (5,6-difluoro-4,7-dimethylbenzo[*c*][1,2,5]thiadiazole), which contains two fluorine atoms. WLK-6 has an *E*_gap_ value of 3.853 eV, which indicates the impact of the π-spacer (4,7-dimethyl-[1,2,5]thiadiazolo[3,4-*c*]pyridine (K-6)), on minimizing the energy gap. The effect of additional spacers on the energy gap follows this pattern: K-2, K-3, K-4, K-5, K-1, and K-6. Thiazole-based spacers with more electronegative atoms show higher resonance and conjugation than thiophene-containing spacers. Overall, the *E*_gap_ values of all examined compounds are in ascending order: WL-7 > WLK-2 > WLK-3 > WLK-4 > WLK-5 > WLK-1 > WLK-6. This decreasing trend shows that all proposed chromophores have smaller *E*_gap_, which may lead to longer wavelengths and make them excellent candidates for improved NLO and photovoltaic capabilities.

### 3.3 Population analysis

To perform population analysis, we used the PyMOlyze-1.1 software.^55^ To verify the results of the FMOs analysis, a population analysis (DOS) was conducted (see Fig. 4).^56^ To facilitate this analysis, the investigated compounds (WL-7 and WLK-1–WLK-6) were divided into four segments: donor, π-spacer, auxiliary spacer, and acceptors. Fig. 4 illustrates the graphical representation of the TDOS, offering further empirical support and improving observation of the electrical configuration of the compounds under investigation. Within the pictorial illustration, the LUMO or conduction band is depicted by positive values, whereas the HOMO or valence band is represented by negative values.

The energy gap, indicating the distance between the HOMO and LUMO, is displayed on the *x*-axis. Analysis of FMOs revealed that the charge transport pattern on molecular orbitals could be modified by adding specific electron-deficient groups. Table S2 (ESI)† illustrates the percentage density of states associated with the LUMO and HOMO. The reference compound WL-7 comprises four major influence units: donor, spacer, and acceptor. While an auxiliary acceptor is also present, it has little influence in FMO contour plots, so we neglect that part. The calculated charge population in the HOMO from these segments is 19.8% donor, 72.8% spacer, and 7.4% acceptor. In the LUMO, the donor contributes 50%, the spacer 12.1%, and the acceptor 37.9%, respectively. These contributions are consistent with the results of FMO contour plots, which demonstrate a greater charge presence on the spacer and donor fragment in the HOMO as well as in LUMO, respectively.

The electronic contributions of the donor segment in the HOMO for the designed compound WLK-1 to WLK-6 after reference modification are as follows: 74.4%, 68.4%, 62.8%, 68.8%, 73.8%, and 72.2%, whereas the contributions to the LUMO are 4.7%, 1.4%, 1.6%, 1.9%, 5%, and 9.5%, respectively. Similarly, for WLK-1–WLK-6, the π-spacer's contributions at the HOMO are 4.3%, 1.8%, 23.2%, 1.8%, 2%, and 3.2%, while at the LUMO, they are 67.1%, 34.9%, 25.1%, 31.7%, 13.4%, and 12.4%, respectively. The acceptor segment showed participation for the studied compounds at the HOMO as 19.9%, 13.8%, 11%, 14.1%, 17.6%, and 16.7%, whereas the contributions of acceptor fragments to the LUMO are 13.5%, 42.6%, 40%, 36.1%, 23%, and 10.6%, respectively. The present investigation focuses on modifications with auxiliary spacers. These auxiliary spacers contribute approximately 1.3%, 16%, 3%, 15.3%, 6.6%, and 7.8% in the HOMO and 14.7%, 21.1%, 33.3%, 30.2%, 58.6%, and 67.5% in the LUMO. The examination of the proposed compounds (WL-7 and WLK-1–WLK-6) uncovers a notable finding: a significant charge transfer from the electron-rich donor to the electron-withdrawing end-capped acceptor moiety *via* the π-bridges and existence of charge delocalization. This occurrence remains uniform among all the studied compounds, setting the basis for effective charge transfer.

### 3.4 Natural bond orbital (NBO) analysis

This method is frequently employed in computational chemistry to elucidate the nature of chemical bonding within molecules. The approach relies on the principles of hybridization and the dispersion of electrons across different orbitals.^57^ To analyze a molecule's electrical structure, the approach integrates molecular orbital theory with naturally localized molecular orbitals. The calculations shown in Table S3 (ESI)† are based on the NBO examination of numerous bonds in different chemical configurations. The *E*^(2)^ values represent the stabilization energy caused by interactions between donor and acceptor orbitals. A higher *E*^(2)^ value suggests a more robust interaction between these orbitals. The *E*^(*j*)^ values signify the delocalization energy of electron density from a donor orbital to an acceptor orbital. *E*^(*i*)^ values, on the other hand, indicate the occupancy of either the donor or acceptor orbital. Lastly, the *F*_*i*,*j*_ values represent Fock matrix elements that depict the fractional weights of the donor and acceptor orbitals within the NBO framework.^58,59^ This extensive research sheds light on the type and strength of chemical interactions within molecules. The values in Table S3† illustrate a range of orbital interactions such as anti-bonds, pi or sigma bonds, and lone pairs. The NBO analysis of the dyes revealed several significant values: *E*^(2)^ represents the stabilization energy, *E*^(*j*)^ signifies donor–acceptor interaction energy, *E*^(*i*)^ (a.u.) indicates lone pair interaction energy, and *F*_*i*,*j*_ (a.u.) denotes hyper-conjugative interaction energy. The values above impart crucial insights regarding dye molecules' stability and electrical interaction, essential for their non-linear optical (NLO) and photovoltaic (PV) capabilities. Multiple transitions were identified in the NBO study for all dyes (refer to Table S3†). For example, about WL-7, the transition from O 59 (LP) to C57–O58(σ*) has an *E*^(2)^ value of 39.21 kcal mol^−1^, indicating substantial stabilization energy. This transition is crucial in facilitating the efficient separation and movement of charges within the dye molecule. In examining numerous dyes, such as WL-7 and WLK-1 to WLK-6, discrete transitions between certain orbitals were identified, each with different stabilization energies (*E*^(2)^ values) that influence charge separation and energy transfer inside the dye molecules. WLK-1's transition between C23(LP) and C27–O35(π*) has a greater *E*^(2)^ value of 116.86 kcal mol^−1^, indicating robust stabilization. The transition between C13–C14(π) and C2–C10(π*) indicates a reduced stabilization energy required for photon absorption and subsequent charge separation. WLK-1's higher stabilization energy is related to the addition of the thiazole-based K1 extra spacer. In WLK-2, the transition between C23(LP) and C20–C22(π*) has an *E*^(2)^ value of 71.23 kcal mol^−1^, while the transition from C2–C14(π) to C12–C13(π*) has a stabilization energy of 34.1 kcal mol^−1^, indicating considerable stabilization. These transitions enable more efficient energy transmission and charge separation. Similarly, WLK-3 has a high stabilization energy (71.13 kcal mol^−1^) at the transition from C23(LP) to C20–C22(π*). WLK-4 has a high stabilization energy (69.9 kcal mol^−1^) in the transition between C38 (LP) and C36–C37 (π*). In WLK-5 and WLK-6, the transition between C2–C14 (π) and C12–C13 (π*) yields *E*^(2)^ values of 35.25 and 38.08 kcal mol^−1^, respectively, showing significant stabilization energies necessary for energy transfer and charge separation. These transitions and accompanying values provide useful information about the dyes' electronic interactions and stability, critical for their photovoltaic (PV) and NLO response.

### 3.5 Electron excitation analysis

Electronic transition analysis, such as examining transition density matrix (TDM) and hole–electron overlap analysis, plays a crucial role in NLO responses. It helps in recognizing excited states, defining transition, *µ*_tot_, assessing charge transfer, creating NLO materials, and establishing relationships between structure and properties. This study provides insights into the essence and origins of electronic transitions, allowing for more accurate predictions and optimization of NLO behaviour.

#### 3.5.1 Hole–electron overlap analysis

**Fig. 6:**
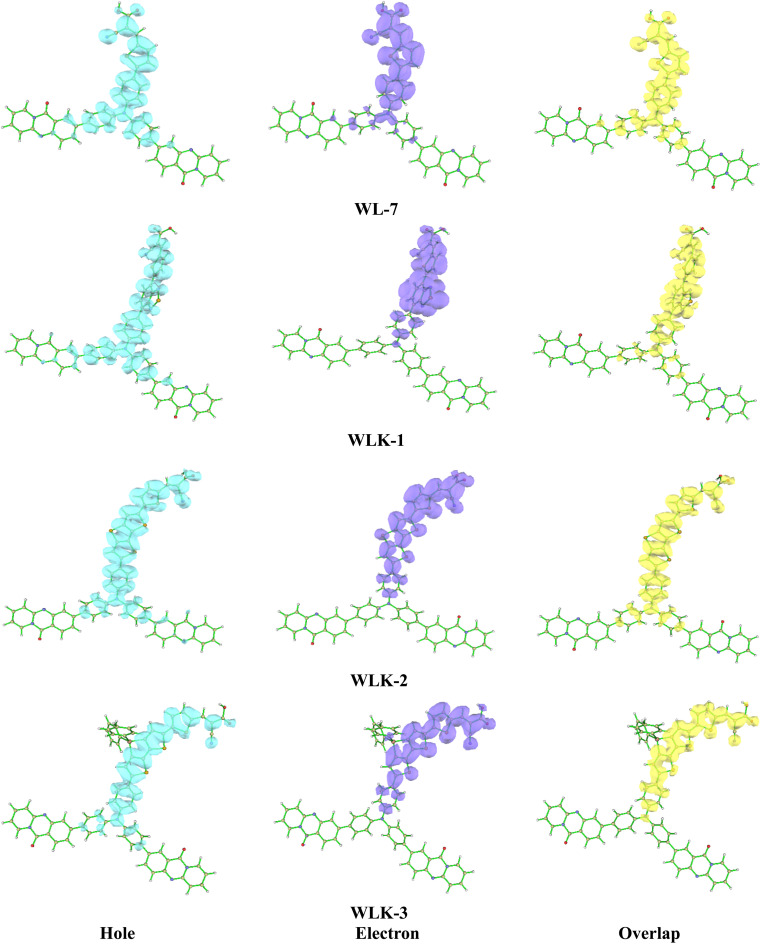
The electron–hole overlap of designed molecules (continued in Fig. S2†).

The analysis of the overlap between holes and electrons in the suggested chromophores offers valuable insights into their electrical properties and possible applications. This inquiry is concentrated on the spatial arrangement of holes (vacancies of positive charge) and electrons in chromophores, a factor that significantly influences their ability to transfer charges and overall electronic performance.^60^ The predominant localization of holes in the examined chromophores was observed to be primarily situated around the donor component and the nitrogen acceptor. Specifically, among the chromophores investigated, chromophores WLK-1 and WLK-6 have the largest hole dispersion (see Fig. 6 and S2†). This finding implies that these compounds have a greater ability to transfer positive charges (holes) inside their molecular structures. Understanding the distribution of holes and electrons in chromophores is crucial for improving performance in nonlinear optics, photonics, and optoelectronics. This analysis aids in leveraging the charge transfer capabilities of chromophores to build new materials with specific electronic functionality. The study compared the overlap of hole and electron distributions in these chromophores, showing a similarity to their charge distributions. This discovery shows that the appropriate spatial overlap of holes and electrons facilitates charge transfer mechanisms within chromophores. The heat map illustration presented in Fig. S3† illustrates the spatial overlap of hole and electron distributions in the chromophores. This comprehensive investigation plays a crucial role in the anticipation and development of chromophores possessing enhanced capabilities for charge transfer, which are essential for their utilization in photovoltaic systems, photocatalytic processes, and optoelectronic devices.^61^ Researchers can alter the molecular architectures of chromophores to optimize charge transfer efficiency, hence improving the performance and usefulness of materials utilized in various technological applications.

#### 3.5.2 TDM analysis

**Fig. 7:**
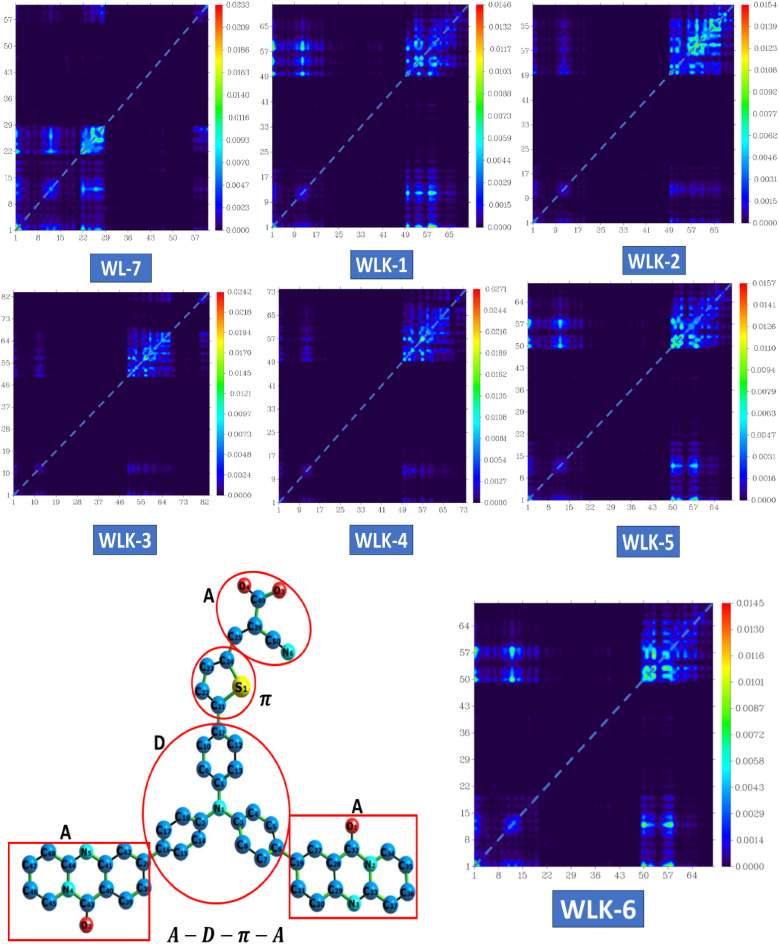
The heat maps of the transition density matrix for all the designed compounds.

The transition density matrix (TDM) of three-dimensional pyridoquinazolindone-containing triphenylamine-based push–pull chromophores was computed to understand better the nature and behaviour of transitions in the excited state.^58^ This study utilizes information about transferring charges from the donor to the acceptor component through π-linkers. The TDM analysis of the proposed compounds shows effective charge propagation from the donor to the acceptor side, with low charge utilization by the π-bridge. To attain this goal, compounds such as WL-7 and WLK-1 to WLK-6 were examined utilizing the aforementioned methodology. This TDM study is important for understanding how charge transfer occurs within these chromophores, notably the involvement of the donor, acceptor, and π-linkers in promoting efficient electronic transitions. Such insights are critical for improving the design and performance of these materials in fields such as photovoltaics and optoelectronics. The atoms were separated into three segments depending on their contributions: donor (D), π-spacers (π), and acceptor (A). Hydrogen atoms were removed due to their minimal participation in effective charge transfer processes. Fig. 7 shows the results for WL-7 as well as WLK-1 to WLK-6. Fig. 7 shows that the electronic charge densities of these chromophores are primarily localized along the diagonal from the donor (D) to the π-linkers (π). This diagonal charge transfer pattern is similar in all push–pull chromophores, showing effective charge transfer over the π-bridge from the donor to the acceptor segment and minimizing charge entrapment. The bright spots in the spectrum correspond to certain atoms found in the proposed compounds. For WL-7 and WLK-1 to WLK-6 (excluding WLK-3), significant electronic transitions are detected at atom numbers 1–19, corresponding to the donor fragments, and atom numbers 50–97, corresponding to the auxiliary spacer and π-spacer segments. For WLK-3, it was found that the donor atoms carry a slight charge, indicating their crucial involvement in the primary electronic transitions occurring within these chromophores. Analysis of the transition density matrix (TDM) indicates that these transitions greatly influence the optical properties of WL-7 and WLK-1 to WLK-6. Understanding and regulating these electronic transitions is critical for designing nonlinear optics, photonics, and photovoltaics applications.^56,58^ Researchers may use TDM analysis findings to optimize the design of these chromophores, enhancing their performance in various technological applications, including improving efficiency and functionality in optical and energy-related devices.

### 3.6. Non-covalent interaction

The chains in push–pull conjugated materials consist of alternating donor (D) and acceptor (A) systems.^62^ This system demonstrates D–A interaction, also known as intramolecular charge transfer. The narrow HOMO–LUMO energy gaps of donor–acceptor conjugated materials facilitate effective ICT.^63^

#### 3.6.1 NCI-RDG analysis

NCI (non-covalent interaction) is a visualization index that uses density and its derivatives to detect non-covalent interactions.^64^ It focuses on finding peaks in the reduced density gradient (RDG) at low densities. The following equation^65^ defines the RDG:1
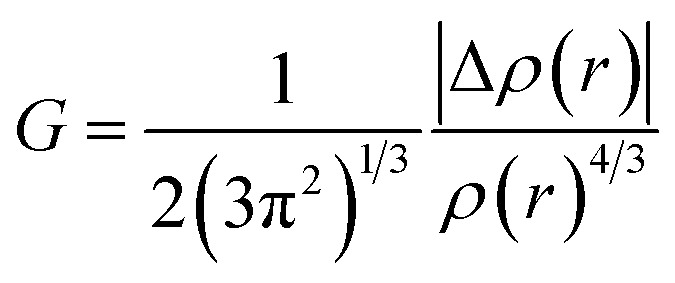


**Fig. 8:**
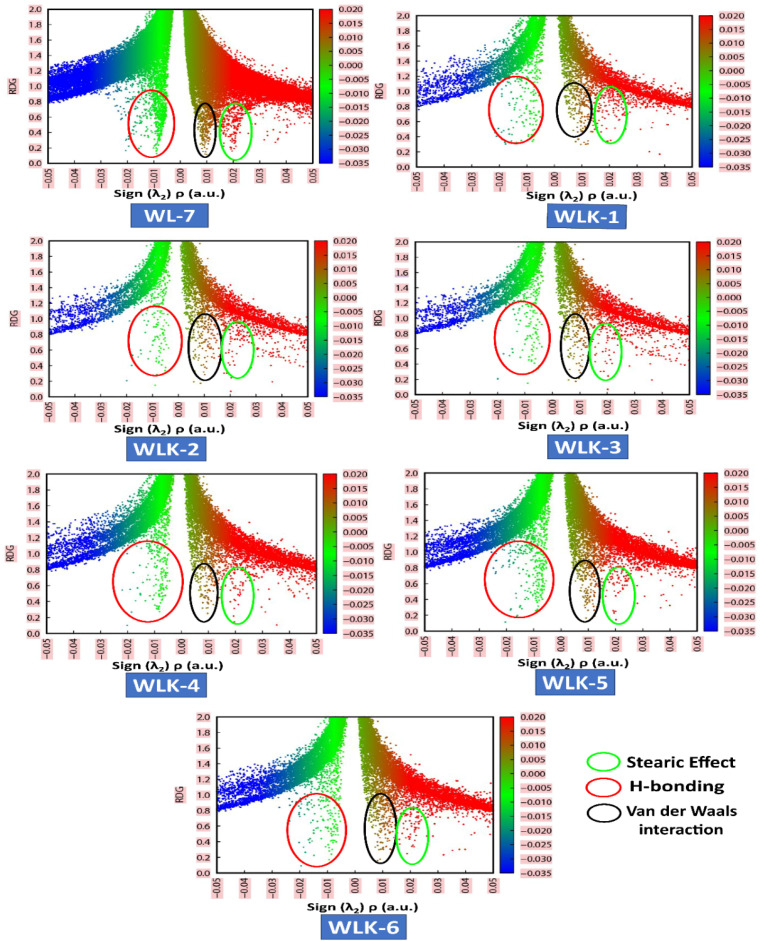
The NCI-RDG plots of all designed compounds.

VMD and Multiwfn software programs were used for the computational analysis. The RDG scatter plot (Fig. 8) shows numerous spikes between 0.05 and 0.5 atomic units. The spikes in green encircling red represent hydrogen bonding (attractive interactions), whereas those in dark green encircling black represent van der Waals (vdW) interactions.

Additionally, spikes encircled in red and surrounded by green represent repulsive interactions, specifically steric effects. The peaks of RDG at approximately 0.031 of sign(*λ*_2_)*ρ* (a.u.) show strong, attractive interactions, supported by the low-density value.^66^ NCI analysis demonstrates that the designed molecules are effective for transferring charge in optical and photovoltaic devices without trapping.

#### 3.6.2 ELF and LOL analysis

The Multiwfn software tool was utilized to conduct analyses on the ELF and LOL. Both ELF and LOL serve as descriptors that offer insights into shell structure, chemical bonding classification, electron localization, and verification of charge-shift bonds on molecular surfaces utilizing kinetic energy density.^67^ Silvi and Savin presented ELF and LOL analysis.^68^ While they have comparable chemical meanings, ELF focuses on electron pair density, whereas LOL looks at the overlap of localized orbital.^69^ Significant ELF values (ranging from 0.5 to 1.0) connected to localized electrons may justify the existence of covalent bonds. Conversely, diminished ELF values (below 0.5) indicate the phenomenon of electron delocalization.^70^ Fig. S4 (ESI)† illustrates the colour-filled maps of LOL and ELF. Within the ELF map, two primary regions are identified, namely blue and red. The blue areas adjacent to molecules such as C, N, O, S, and Si symbolize a delocalized electron cloud, whereas the red regions exhibit elevated ELF values, signifying localized electrons. The colour-filled map displayed by LOL illustrates a white central area encircling hydrogen atoms, signifying an electron density surpassing the upper limit of the colour scale (0.80). Red is assigned to this elevated value, confined between atoms, denoting covalent interactions. Conversely, regions in blue represent low values indicating non-covalent interactions, specifically hydrogen bonds. Understanding and manipulating these transitions provides opportunities for tailored applications in a variety of fields, including molecular electronics, photonic devices, optoelectronics, biomedical imaging and non-linear optics. This optimization can dramatically improve performance within each specific domain.

### 3.7 Electrostatic surface analysis

**Fig. 9:**
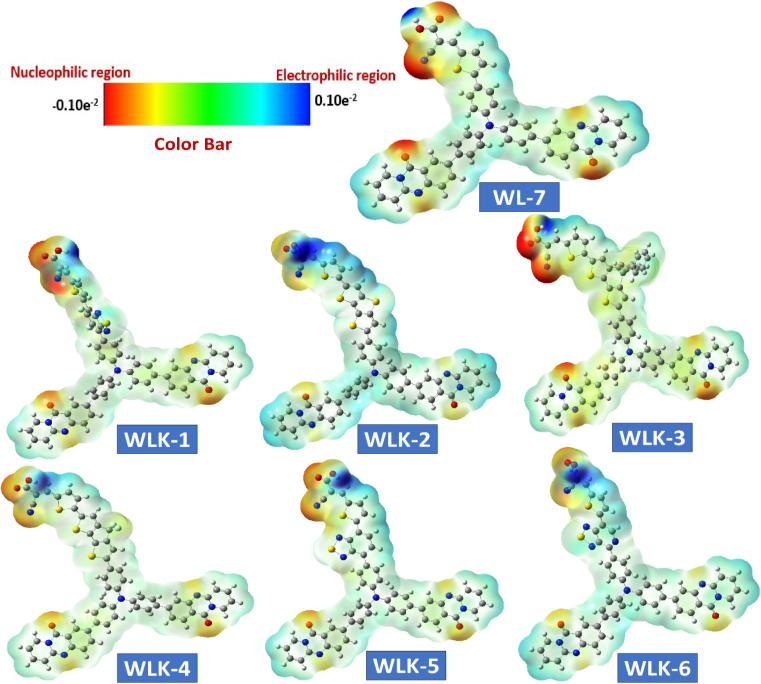
The ESP surface of all designed compounds.

A molecule's electrostatic potential (ESP) gives important information about its structural stability and reveals essential sites vulnerable to interaction with other molecules.^71^Fig. 9 depicts the mapped molecular electrostatic potential (MEP) of modelled molecules, with blue and red indicating significant positive and negative static charge locations, respectively, and quantitative atom-specific data. Based on static charge analysis, Oxygen atoms in all known and modeled compounds have extensive negative charge clouds, which indicate possible reactive sites. Nitrogen atoms in cyanide molecules also have significant negative charge clouds, which serve as reaction sites due to their terminal molecular framework location in the absence of neutralizing atoms. The negatively charged reaction sites in WL-7 and WLK-based dyes are near nitrogen atoms in acceptors and donors. Positively charged reaction sites in WL-7 and WLK-based dyes are found near nitrogen atoms in acceptor and donor regions due to adjacent to carbon atoms cancelling out positive charges. Overall, nitrogen atoms in cyanide molecules and corner carbon atoms in molecular frameworks have a positive charge cloud (blue area) and negative charge cloud (red area), respectively, demonstrating different charge properties critical for understanding molecular interactions and reactivity.

### 3.8 Photophysical properties

**Fig. 10:**
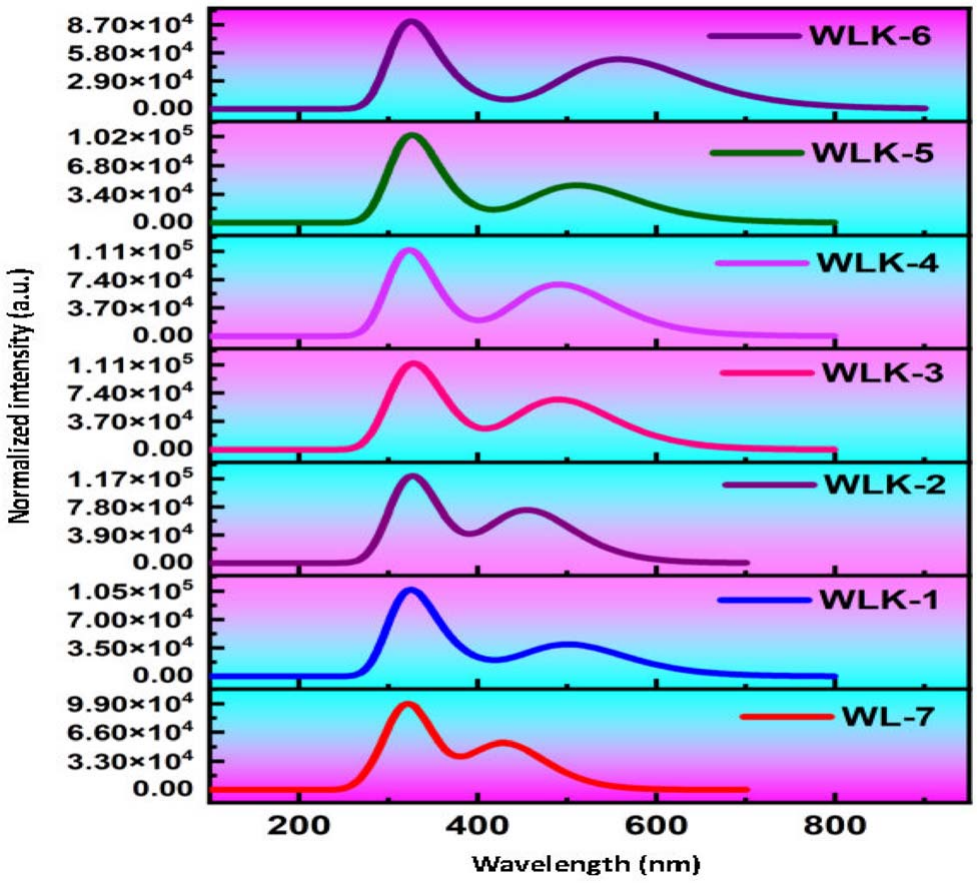
The simulated absorption spectra of all designed compounds.

The primary objective is to analyze how modifications to the π-spacer influence the photophysical properties of WL-7 and WLK-1 to WLK-6 through TD-DFT calculations utilizing the CAM-B3LYP functional with a 6-311G(d,p) basis set, excited state absorption spectra were produced. The five lowest singlet–singlet transitions were estimated using the CPCM model and a methanol solvent.^72^Table 1 summarizes the computed results of oscillator strength (*f*_os_), absorption wavelength (*λ*_max_), transition energy (*E*_ext_), and the nature of transitions for the studied substances. Notably, Table 1 shows that all dyes tested have absorbance within the visible spectrum. WL-7 has the shortest wavelength of all the compounds investigated, with an absorption maximum (*λ*_max_) of 429.844 nm (experimental *λ*_exp_ = 428 nm). These systems' *λ*_max_ values are strongly correlated with their structural properties. Thus, as shown in Fig. 10, altering the structure of WL-7 by adding various extended π-spacers (K-1 to K-6) to improve the π-conjugation of the thiazole-based first spacer affects the *λ*_max_ value. When π-spacers are added to WLK-6, WLK-5, and WLK-1 compared to WL-7, the absorption wavelength improves and shifts towards longer wavelengths in the 400–600 nm range. Incorporating π-bridges K2, K3, and K4 in WLK-2, WLK-3, and WLK-4 results in a lower absorption wavelength, with *λ*_max_ values ranging from 250–400 nm. The maximum recorded *λ*_max_ value is 558.613 nm for WLK-6, which is 118 nm longer than the standard WL-7. These spectral results show that modified compounds absorb near-infrared light absorption after structural alterations.

**Table 1:** The photophysical parameters calculated at CAM-B3LYP/6-31G(d,p) level of theory

Molecules	*E*_ext_	*λ*_max_	*f*_os_	Major electronic transitions
WL-7	2.884	429.844	1.3138	H−3 → LUMO (12%), HOMO → LUMO (73%)
WLK-1	2.467	502.489	0.9638	H−3 → LUMO (15%), HOMO → LUMO (69%)
WLK-2	2.721	455.606	1.8067	H−3 → LUMO (12%), H−1 → LUMO (32%), HOMO → LUMO (42%)
WLK-3	2.526	490.813	1.6106	H−1 → LUMO (33%), HOMO → LUMO (49%)
WLK-4	2.526	490.890	1.6658	H−3 → LUMO (11%), H−1 → LUMO (28%), HOMO → LUMO (43%)
WLK-5	2.425	511.191	1.0975	H−3 → LUMO (16%), H−1 → LUMO (11%), HOMO → LUMO (65%)
WLK-6	2.219	558.613	1.2657	HOMO → LUMO (76%)

The *λ*_max_ values in decreasing order are as follows: WLK-6 (*λ*_max_ = 558.613 nm) > WLK-5 (*λ*_max_ = 511.191 nm) > WLK-1 (*λ*_max_ = 502.489 nm) > WLK-4 (*λ*_max_ = 490.890 nm) > WLK-3 (*λ*_max_ = 490.813 nm) > WLK-2 (*λ*_max_ = 455.606 nm) > WL-7 (*λ*_max_ = 429.844 nm). The increased absorption wavelength in the S0 → S1 transitions of the designed dye is linked to the impact of the added π-spacers incorporated into the molecular structure. Furthermore, the transitions observed in WL-7 and WLK-1 to WLK-6 primarily come from HOMO to LUMO, which is consistent with the normal behaviour seen in most Donor–π–Acceptor organic chromophores. HOMO → LUMO interactions dominate these transitions, accounting for 40–76% of their intensity. HOMO−3 → LUMO contributes 10–16%, while HOMO−1 → LUMO contributes 11–33% from WLK-2 to WLK-5. The HOMO → LUMO (π–π*) transition accounts for the majority of the highest absorption peaks in all compounds. The substitution of π-bridges within WL-7 led to enhancements in the absorption wavelength of the dyes, rendering them possible candidates for future new dyes.

### 3.9 Non-linear optical properties

#### 3.9.1 Solvent assisted NLO

Nonlinear optics (NLO) has sparked tremendous scientific interest due to its potential to generate transformative advancements in various crucial applications. This field possesses the potential to revolutionize optical technology by facilitating advancements in frequency shifting, molecular switching, optical modulation, memory, and the development of optical fibre material logic. The distinctive non-linear properties of specific materials, alongside their capacity to manipulate light in innovative manners, underscore their significance in the progress of optical science and technology. NLO phenomena arise from the interaction of intense, coherent radiation beams with substances. High-quality NLO materials exhibit enhanced hyperpolarizability values.^73^ Organic nonlinear optical (NLO) materials exhibit distinctive nonlinear characteristics caused by delocalized electrons within the π → π* orbitals. The calculation of the static dipole moment (*µ*_tot_), linear polarizabilities (*α*_ISO_ and *α*_ANISO_), and first-order hyperpolarizability (*β*_0_) were conducted using the CAM-B3LYP/6-311G(d,p) approach. Tables 2–4 summarize the results. The static dipole moment's *x*, *y*, and *z* components can be expressed by the following equation:2
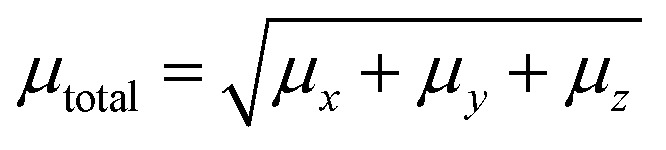


**Table 2:** Solvent-dependent dipole moment in D. for WL-7, and WLK-1 to WLK-6

Molecules	Gas phase	Methanol	DMSO	THF	Benzene	Water
WL-7	5.07	6.87	6.91	6.54	5.85	6.94
WLK-1	1.06 × 10^1^	1.32 × 10^1^	1.32 × 10^1^	1.27 × 10^1^	1.17 × 10^1^	1.33 × 10^1^
WLK-2	1.00 × 10^1^	1.31× 10^1^	1.31 × 10^1^	1.25 × 10^1^	1.13 × 10^1^	1.32 × 10^1^
WLK-3	1.23 × 10^1^	1.62 × 10^1^	1.63 × 10^1^	1.55 × 10^1^	1.40 × 10^1^	1.63 × 10^1^
WLK-4	9.02	1.12 × 10^1^	1.13 × 10^1^	1.08 × 10^1^	9.95	1.13 × 10^1^
WLK-5	1.14 × 10^1^	1.51 × 10^1^	1.52 × 10^1^	1.44 × 10^1^	1.29 × 10^1^	1.53 × 10^1^
WLK-6	1.08 × 10^1^	1.34 × 10^1^	1.35 × 10^1^	1.29 × 10^1^	1.19 × 10^1^	1.35 × 10^1^

**Table 3:** Solvent-dependent linear polarizability in a.u. for WL-7 and WLK-1 to WLK-6

Molecules	Gas phase	Methanol	DMSO	THF	Benzene	Water
WL-7	7.34 × 10^2^	5.55 × 10^2^	5.57 × 10^2^	5.34 × 10^2^	8.22 × 10^2^	5.59 × 10^2^
WLK-1	5.33 × 10^2^	6.86 × 10^2^	6.89 × 10^2^	6.58 × 10^2^	5.99 × 10^2^	6.92 × 10^2^
WLK-2	5.73 × 10^2^	7.18 × 10^2^	7.21 × 10^2^	6.92 × 10^2^	6.37 × 10^2^	7.24 × 10^2^
WLK-3	6.32 × 10^2^	8.03 × 10^2^	8.06 × 10^2^	7.71 × 10^2^	7.06 × 10^2^	8.09 × 10^2^
WLK-4	5.66 × 10^2^	7.07 × 10^2^	7.09 × 10^2^	6.81 × 10^2^	6.28 × 10^2^	7.12 × 10^2^
WLK-5	4.99 × 10^2^	6.37 × 10^2^	6.39 × 10^2^	6.11 × 10^2^	5.59 × 10^2^	6.42 × 10^2^
WLK-6	5.51 × 10^2^	7.14 × 10^2^	7.17 × 10^2^	6.84 × 10^2^	6.22 × 10^2^	7.20 × 10^2^

**Table 4:** Solvent-dependent first hyperpolarizability in a.u. for WL-7 and WLK-1 to WLK-6

Molecules	Gas phase	Methanol	DMSO	THF	Benzene	Water
WL-7	1.75 × 10^4^	4.06 × 10^4^	4.10 × 10^4^	3.63× 10^4^	2.73 × 10^4^	4.14 × 10^4^
WLK-1	3.72 × 10^4^	5.89 × 10^4^	5.91× 10^4^	5.67× 10^4^	4.94 × 10^4^	5.92 × 10^4^
WLK-2	3.59 × 10^4^	6.44 × 10^4^	6.48× 10^4^	6.04× 10^4^	4.99 × 10^4^	6.51 × 10^4^
WLK-3	3.85 × 10^4^	7.80 × 10^4^	7.86× 10^4^	7.20× 10^4^	5.69 × 10^4^	7.91 × 10^4^
WLK-4	4.45 × 10^4^	7.63 × 10^4^	7.67× 10^4^	7.21× 10^4^	6.07 × 10^4^	7.70 × 10^4^
WLK-5	3.83 × 10^4^	6.88 × 10^4^	6.92× 10^4^	6.44× 10^4^	5.32 × 10^4^	6.96 × 10^4^
WLK-6	6.25 × 10^4^	1.22 × 10^5^	1.23× 10^5^	1.12× 10^4^	9.00 × 10^4^	1.23 × 10^5^

The following equation of linear polarizability [99] describes the isotropic and anisotropic features of designed chromophores:3
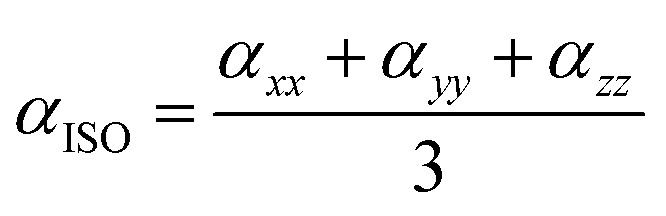
4
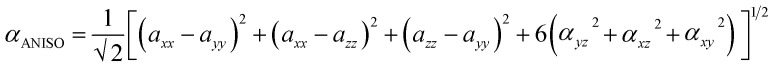


A 3 × 3 × 3 matrix defines the first-order hyperpolarizability, a third-degree tensor. Kleinman's symmetry reduces the 27 individual elements found in the three-dimensional matrix to a total of 10 distinct components. The following equation can help in the calculation:

5 *β*_0_ = [(*β*_*xxx*_ + *β*_*xyy*_ + *β*_*xzz*_)^2^ + (*β*_*xxy*_ + *β*_*yyy*_ + *β*_*yzz*_)^2^ + (*β*_*xxz*_ + *β*_*zyy*_ + *β*_*zzz*_)^2^]^1/2^

Traditionally, nonlinear optical (NLO) features in the gas phase have been approximated theoretically.^74–77^ However, condensed phases are usually included in experimental evaluations of NLO characteristics, where solvent effects become important. Through its interactions with the solute, the solvent can modify the characteristics of NLO. Density functional theory (DFT) computations on molecules WL-7 and WLK-1 to WLK-6 in the gas phase were carried out to study this, utilizing the B3LYP/6-31G(d,p) basis sets. Furthermore, the polarizable continuum model (IEF-PCM) with integral equation formalism was utilized to investigate the influence of medium polarity. We investigated first hyperpolarizability as well as polarizability in response to several solvents whose dielectric constants (*ε*) ranged from 2.27 to 78.36. These solvents covered a diverse spectrum of medium polarity, ranging from the least polar to the most polar, which included water (*ε* = 78.36), methanol (*ε* = 32.61), DMSO (*ε* = 46.83), tetrahydrofuran (*ε* = 7.43), benzene (*ε* = 2.27) and chloroform (*ε* = 4.71). The availability of a wide range of solvents enabled us to assess the influence of medium polarity on the nonlinear optical performance of the designed molecule.

Tables 2–4 show the nonlinear optical parameters computed in the gas and solvent phases were compared to those of urea, which acts as a reference molecule for the evaluation of NLO properties (*µ*_urea_ = 1.373 D, *α*_urea_ = 5.047 × 10^−24^ esu (5.84 × 10^8^ a.u.), and *β*_urea_ = 372.8 × 10^−33^ esu (4.32 × 10^1^ a.u.)).^78,79^ The total dipole moments (*µ*_tot_) of WL-7 and WLK-1 to WLK-6 were determined in the following sequence: WLK-3 > WLK-5 > WLK-6 > WLK-1 > WLK-2 > WLK-4 > WL-7. Importantly, the dipole moments (*µ*_tot_) of all substances examined surpass those of urea. Each proposed compound has a dipole moment ranging from 5 to 16 D. Upon measuring the dipole moments in polar solvents (water, THF, methanol, and DMSO) and a non-polar solvent (benzene), it was observed that the dipole moment values increase in the solvent phase compared to the gas phase. Both in the gas and solvent phases, WLK-3 containing K2 (2,6-dimethyl-4,4-diphenyl-4*H*-silolo[3,2-*b*:4,5-*b*′]dithiophene) exhibited the highest dipole moment, while WL-7, which has a less conjugated π-core, showed the least dipole moment. Furthermore, it was noted that substituting electronegative atoms on the thiadiazole and thiophene-based spacer leads to increased polarization. The predicted effect due to electron-withdrawing atoms follows this order: Si > F > N.

The calculations reveal that molecules WLK-1 to WLK-6 exhibit notable levels of both isotropic and anisotropic behavior. The average linear polarizability (*α*_0_) defines the linear response, and it is of interest to explore the impact of various π-linkers on *α*_0_ and their role in shaping the structural nonlinear optical properties in the presence of an electric field. The linear polarizability (*α*_0_) values of the examined compounds were sorted in ascending order for evaluation in the gas phase, and non-polar solvent (benzene) are as follows: WL-7 > WLK-3 > WLK-2 > WLK-4 > WLK-6 > WLK-1 > WLK-5. In polar solvents (water, DMSO, methanol, and THF), the linear polarizability (*α*_0_) values shift to WLK-3 > WLK-2 > WLK-6 > WLK-4 > WLK-1 > WLK-5 > WL-7. The proposed molecules' high average polarizability (*α*_0_) values show a well-balanced distribution of electrical charges. Despite the non-centrosymmetric character of the materials under investigation, Table 3 shows that the inclusion of a terthiophene linker with a Si atom and benzene substitution allows the WLK-3 compound to have higher static polarizability values than other investigated dyes.

Furthermore, the initial hyperpolarizability (*β*_0_) values were roughly 6000 times higher than the standard urea. The greater hyperpolarizability values are directly linked to the ICT characteristics of the electron-donating and electron-withdrawing groups. Table 4 demonstrates that the proposed push–pull systems exhibit high hyperpolarizability (*β*_0_) and are suitable for NLO response. The magnitude of the electron–donor pair's strength is specifically associated with an increase in the first hyperpolarizability values. The π-linkers connecting electron-donating and accepting groups facilitate conjugation, leading to electron delocalization and elevated static first hyperpolarizability (*β*_0_) values, following a decreasing sequence: WLK-6 > WLK-4 > WLK-3 > WLK-5 > WLK-1 > WLK-2 > WL-7. When compared to the standard WL-7, WLK-6 has higher static first hyperpolarizability than WLK-4, WLK-3, WLK-5, WLK-1, WLK-2, and WL-7. The static first hyperpolarizability values (*β*_0_) for the WLK-6 molecule with the thiophene ring and 4,7-dimethyl-[1,2,5]thiadiazolo[3,4-*c*]pyridine (K-6) as the π-linker are as follows: 1.23 × 10^5^ (DMSO), 1.23 × 10^5^ (water), 1.22 × 10^5^ (methanol), 1.12 × 10^5^ (THF), 9.00 × 10^4^ (benzene), and 6.25 × 10^4^ (gas).

#### 3.9.2 Frequency-dependent NLO

**Fig. 11:**
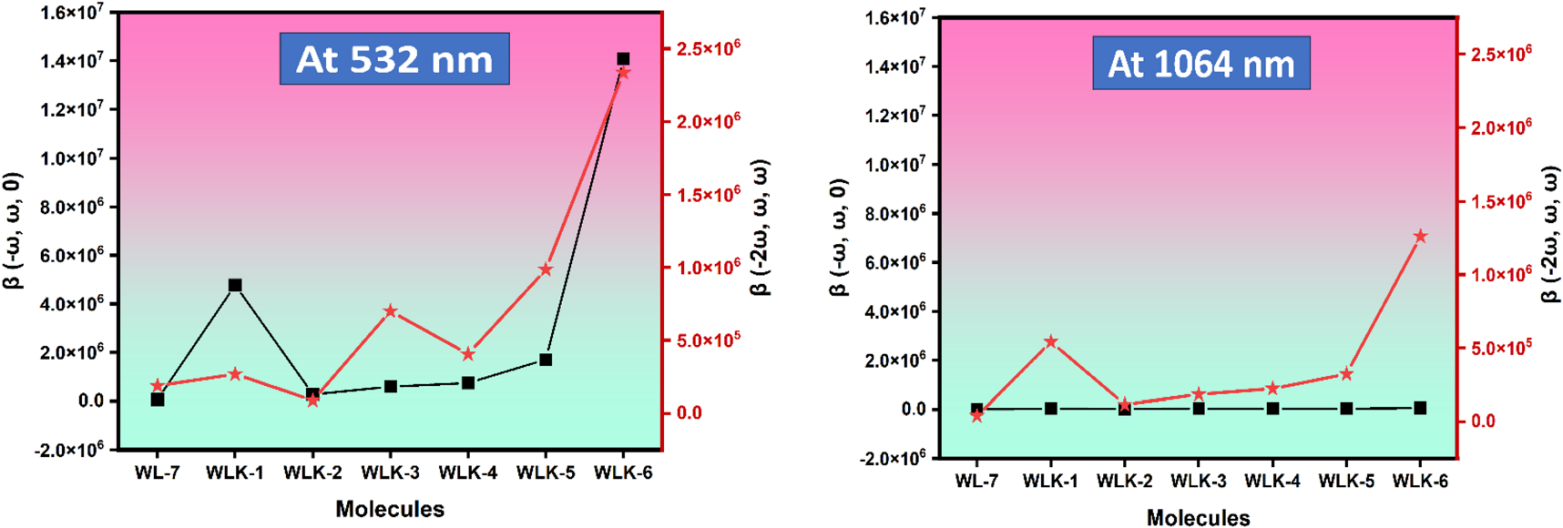
The graphical representation of the frequency-dependent hyperpolarizability.

The impact of frequency on the NLO characteristics of the analyzed chromophores was assessed using two standard frequencies associated with Nd:YAG laser: *ω* = 0.0428 a.u. (1064 nm) and *ω* = 0.0856 a.u. (532 nm). Frequency-dependent calculations were carried out to investigate the electro-optical Pockels effect (EOPE) *β*(−*ω*, *ω*, 0) and second harmonic generation (SHG) *β*(−2*ω*, *ω*, *ω*), which are two significant first hyperpolarizability coefficients.^80^ Table S4† shows the values for EOPE and SHG. The results of both EOPE and SHG studies show that the nonlinear optical responses of the investigated compounds are strongly impacted by frequency and can be controlled by an applied magnetic field. Table 4 shows that all chromophores' static first hyperpolarizability (*β*_0_) values range from 3.75 × 10^4^ to 1.05 × 10^4^ atomic units (a.u.). The EOPE values (*β*(−*ω*, *ω*, 0)) of our compounds at 1064 nm vary from 6.65 × 10^4^ to 1.48 × 10^4^ a.u. The EOPE values vary from 1.41 × 10^7^ to 7.30 × 10^4^ a.u. at 532 nm (Fig. 11).

The WLK-6 compound exhibits the highest values of EOPE and SHG at 532 nm and 1064 nm, primarily attributed to the presence of the K6 spacer, which aligns well with the static first hyperpolarizability. At 532 nm, the trend for EOPE and SHG values, in ascending order, is observed as follows: WLK-6 > WLK-1 > WLK-5 > WLK-4 > WLK-3 > WLK-2 > WL-7 for EOPE, and WLK-6 > WLK-5 > WLK-3 > WLK-4 > WLK-1 > WL-7 > WLK-2 for SHG. Similarly, at 1064 nm, the ascending order of EOPE and SHG values is as follows: WLK-6 > WLK-4 > WLK-1 > WLK-5 > WLK-3 > WLK-2 > WL-7 for EOPE, and WLK-6 > WLK-1 > WLK-5 > WLK-4 > WLK-3 > WLK-2 > WL-7 for SHG. These results reveal that the compounds that have been designed have significant frequency-dependent nonlinear optical (NLO) capabilities in the transparent area and that WLK-6 performs very well at both wavelengths.

### 3.10 Photovoltaic properties

Photovoltaic attributes play a crucial role in investigating and advancing solar cells. The subsequent are essential photovoltaic features that are commonly analyzed and discussed.

#### 3.10.1 Open circuit voltage (*V*_OC_)

The following equation can be used to compute the energy difference between the lowest unoccupied molecular orbital (LUMO) and the energy of the conduction band (*E*_CB_) of WL-7 molecules:^81^

6 *V*_OC_ = *E*_LUMO_ − *E*_CB_

Table S5 (ESI)† shows the computed V_OC_ values for WL-7 and WLK-1 to WLK 6. All investigated compounds had *V*_oc_ values ranging from 2.361 to 1.442 eV. It is worth noting that all *V*_oc_ values are positive, indicating that electrons can easily flow from the dye to the compound's *E*_CB_.

#### 3.10.2 Light harvesting efficiency (LHE)

“light harvesting efficiency” (LHE) refers to a material's ability to capture and convert incoming light into electrical current. It is generally defined as the ratio of charge carriers (*e.g.*, electrons and holes) produced to photons absorbed across a given wavelength range. LHE is an important component in determining the overall efficiency of a photovoltaic device since it directly determines how much electrical power can be generated from a given amount of incident light. Materials with high LHE can produce more charge carriers per photon absorbed. This property may lead to increased power conversion efficiency in photovoltaic devices.^82^ Essentially, a greater LHE means better utilization of incoming light for electricity generation, which is vital for improving the performance and efficiency of solar cells and other light-harvesting technology.

7 LHE = 1 − 10^−*f*^

Table S5† shows the light-harvesting efficiency (LHE) values for WL-7 and WLK-1 to WLK-6, which range from 0.984 to 0.891 for all compounds investigated. Notably, WLK-2 has the highest LHE value (0.984). This suggests that all of these dyes have the potential to produce a photocurrent response, with WLK-2 being the most efficient at converting absorbed light into electrical current among the compounds studied.

#### 3.10.3 Electron injection efficiency (Δ*G*_ingect_)

To improve short-circuit current density (*J*_SC_) in photovoltaic systems, improve the driving force for electron injection (Δ*G*^inject^) from photo-induced excited states of dye molecules to the material's surface. A greater Δ*G*^inject^ increases the electron injection rate, leading to higher *J*_SC_ values. Calculating the electron injection rate is useful for understanding and optimizing this process. Improving Δ*G*^inject^ can increase photovoltaic device performance, resulting in higher efficiency and efficacy in converting light into electrical current.^83^

8 Δ*G*^inject^ = *E*^dye^* + *E*_CB_

The energy of the conduction band (*E*_CB_) refers to the reduction potential of the conduction band of a material like TiO_2_. On the other hand, *E*^dye^* represents the excited state oxidation potential energy specific to the dye molecule. These terms are essential in understanding the electron transfer processes involved in dye-sensitized solar cells (DSSCs), where the excited state of the dye (*E*^dye^*) must have sufficient energy to inject an electron into the conduction band of the semiconductor material (TiO_2_) to facilitate efficient charge separation and current generation.

9 *E*^dye^* = *E*^dye^_OX_ − *λ*^ICT^_max_

The ground state oxidation potential (*E*^dye^_OX_) is equivalent to the negative of the highest occupied molecular orbital (HOMO) energy (*E*_HOMO_). This potential is important in understanding the energy level of the dye's ground state. The maximum wavelength of intramolecular charge transfer (ICT) (*λ*^ICT^_max_) is influenced by the value of *E*_HOMO_ and the transition energy with the highest oscillator strength. This wavelength provides insight into the electronic transitions within the dye molecule.

Table S5† shows that the energy shift associated with electron injection (Δ*G*^inject^) varies between 0.191 and −0.535 eV for the dyes investigated. This value indicates the driving force behind electron injection from the dye's photo-induced excited state to the semiconductor surface (*e.g.*, TiO_2_) in DSSCs. The Δ*G*^inject^ decreases in the following sequence: WLK-6 > WLK-1 > WLK-5 > WLK-4 > WLK-3 > WLK-2 > WL-7. This trend shows how different dyes have varying abilities in aiding electron injection, with WLK-6 having the strongest driving power among the dyes investigated.

#### 3.10.4 Electron regeneration energy (Δ*G*^regen^)

The impact of dye regeneration on the overall performance of a DSSC is significant. A lower regeneration rate may restrict the amount of electrical energy generated, whereas a higher rate can enhance efficiency.^84^ Dye regeneration plays a crucial role in creating and optimising DSSCs as a photovoltaic factor to consider, with values between 1.578 and 1.295 for WL-7 and WLK-1 to WLK-6. The effectiveness of dye regeneration substantially influences a DSSC's performance, impacting both the quantity of electrical energy produced and the overall efficiency. Therefore, dye regeneration is essential to the development and enhancement of DSSCs. The greatest values are observed for WLK-1.

10 Δ*G*^regent^_dye_ = *E*^electrolyte^_redox_ + *E*^dye^_oxi_

On the other hand, (*E*^electrolyte^_redox_) represents the ground state oxidation potential energy of the dye molecule. This value is crucial for understanding the energy levels of the dye's ground state, particularly in the context of electron transfer processes within DSSCs. The redox potential of the triiodide-iodide couple (*E*^dye^_oxi_) is a critical parameter in DSSCs and is typically valued at 4.85 eV.

#### 3.10.5 Scharber model

**Fig. 12:**
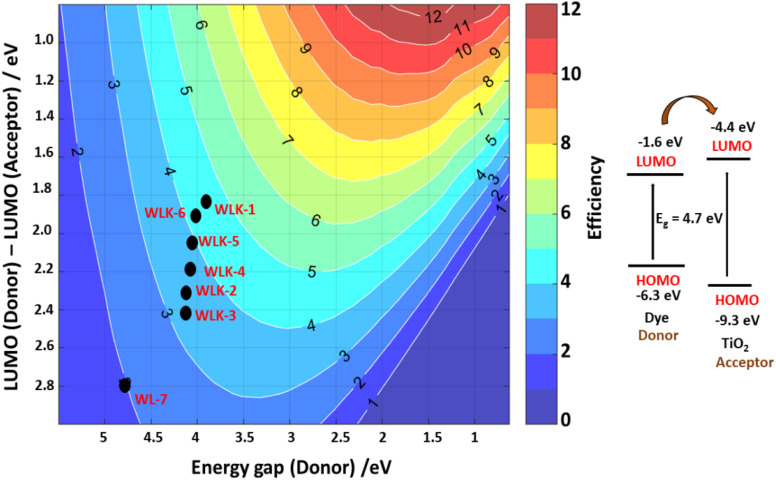
The Scharber diagram shows the predicted photoenergy-conversion efficiency of designed push–pull dyes.

One popular method for estimating the power conversion efficiency (PCE) of donor materials in organic solar cells is the Scharber diagram.^85^ The relationship between the donor material's energy gap and its LUMO energy level is shown in this diagram. As seen in Fig. 12, we have modified this method to predict the PCE of dyes in DSSCs. Based on this investigation, the predicted PCE of the suggested DSSCs ranges from 2% to 5%. Notably, it is anticipated that the reference compound WL-7 will have the lowest PCE, whereas other developed compounds, more especially those containing thiophene, should perform better. Based on the Scharber diagram estimations, WLK-6, WLK-5, and WLK-1 demonstrate the most favourable photovoltaic efficiency out of the analysed compounds. The results indicate that WLK-6, WLK-5, and WLK-1 are promising candidates for creating effective dye-sensitized solar cells, indicating the possibility of raising the total efficiency and effectiveness of DSSC devices.

## 4. Conclusion

Motivated by the need to create new NLO materials with improved static, dynamic, and solvent-dependent NLO properties, as well as by the recognition of their significance for use in solar cell technology, several push–pull pyridoquinazolindone-containing triphenylamine-based chromophores (WLK-1 to WLK-6) with A–D–π–A architecture have been proposed. By conducting a theoretical examination utilizing DFT and its TD-DFT, the study investigates the electronic configuration, transfer of electric charge, solar energy, optical characteristics, and conversion potential of the developed compounds. The calculated results show that the materials under study have very small bandgap energies, between 3.853–4.710 eV. Intermolecular charge transfer is feasible, as demonstrated by the effective electron transfer from the donor (D) to the acceptor (A), which was made possible by the π-conjugated spacer, according to NBO analysis. Context charts from the TDM and electron–hole overlap analysis were also used to support the ICT procedure. To gain a more profound comprehension of the interactions among molecules, we carried out several topological investigations, such as LOL, RDG-NCI, and ELF. Importantly, the dipole moments (*µ*_tot_) of all substances examined surpass those of urea. Each proposed compound has a dipole moment ranging from 5 to 16 D. Upon measuring the linear polarizability (*α*_0_) and first hyperpolarizability (*β*_0_) in polar solvents (water, THF, methanol, and DMSO) and a non-polar solvent (benzene), it was observed that the NLO response increases in the solvent phase compared to the gas phase. For evaluation of *α*_0_ in the gas phase and non-polar solvent (benzene), is as follows: WL-7 > WLK-3 > WLK-2 > WLK-4 > WLK-6 > WLK-1 > WLK-5. In polar solvents (water, DMSO, methanol, and THF), the linear polarizability (*α*_0_) values shift to WLK-3 > WLK-2 > WLK-6 > WLK-4 > WLK-1 > WLK-5 > WL-7. The static first hyperpolarizability (*β*_0_) was elevated from 4.14 × 10^4^ a.u. to 1.23 × 10^5^ a.u. in water (polar) solvent as a result of π-acceptor modification. In addition, the laser-dependent behaviour (iso, aniso *α*(−*ω*, *ω*), EOPE *β*(–*ω*, *ω*, 0) and SHG *β*(–2*ω*, *ω*, *ω*)) of the designed compounds are determined by implying two laser of frequency 0.0856 and 0.0428 utilizing IEFPCM model. WLK-6 with the thiophene ring and 4,7-dimethyl-[1,2,5]thiadiazolo[3,4-*c*]pyridine (K-6) as the π-linker exhibit remarkable highest EOPE and SHG responses at 532 nm and 1064 nm. Similarly, WLK-6 shows maximum recorded values about 558.613 nm. Remarkably, these materials also exhibit optical responses that mostly span the solar spectrum, with light-collecting efficiencies varying from 558.613 to 429.844 nm in methanol solvent. So, to comprehend these optical findings, photovoltaic properties (VOC, LHE, Δ*G*^inject^, and Δ*G*^regent^) of designed compounds are also determined. Scharber's graphic indicates that the projected PCE values are as high as 5%. From the findings, the studied compound's (WLK-3, WLK-6, WLK-1) shows notable *V*_oc_, high LHE, greater optical absorption, small bandgap energy with greater PCE, making them promising photosensitizers for solar energy applications as well as NLO material.

## Data availability

All relevant data are within the manuscript and its ESI file.†

## Conflicts of interest

There are no conflicts to declare.

## Supplementary Material

RA-014-D4RA05290K-s001
