# Supplementary material for: Synergistic charge-transfer dynamics of novel pyridoquinazolindone-containing triphenylamine-based push–pull chromophores: from structural optimization to performance metrics in photovoltaic solar cells and static, dynamic, solvent-dependent nonlinear optical response applications
Source: RSC Adv. 2024 Oct 14;14(44):32482–500. doi: 10.1039/d4ra05290k (PMC11472850; doi:10.1039/d4ra05290k)
Supplement: RA-014-D4RA05290K-s001 [file RA-014-D4RA05290K-s001.pdf]

Supplementary Information (SI) for RSC Advances.
This journal is © The Royal Society of Chemistry 2024

Supporting Information

Synergistic charge-transfer dynamics of novel pyridoquinazolindone-containing triphenylamine-based push-pull chromophores: From structural optimization to performance metrics in photovoltaic solar cells and static, dynamic, solvent-dependent nonlinear optical response applications

Sehar Nadeem,[1] Abida Anwar, [1] Muhammad Usman Khan,*[1] Abrar Ul Hassan,[2] Khalid Abdullah Alrashidi,[3]

[1]*Department of Chemistry, University of Okara, Okara-56300, Pakistan*
[2]*Lunan Research Institute, Beijing Institute of Technology, 888 Zhengtai Road, Tengzhou, 277599, China*
[3]*Department of Chemistry, College of Science, King Saud University, Riyadh 11451, Saudi Arabia*

Corresponding author's E-mail addresses:

- **Dr. Muhammad Usman Khan**
  E-mail ids: usman.chemistry@gmail.com ; usmankhan@uo.edu.pk

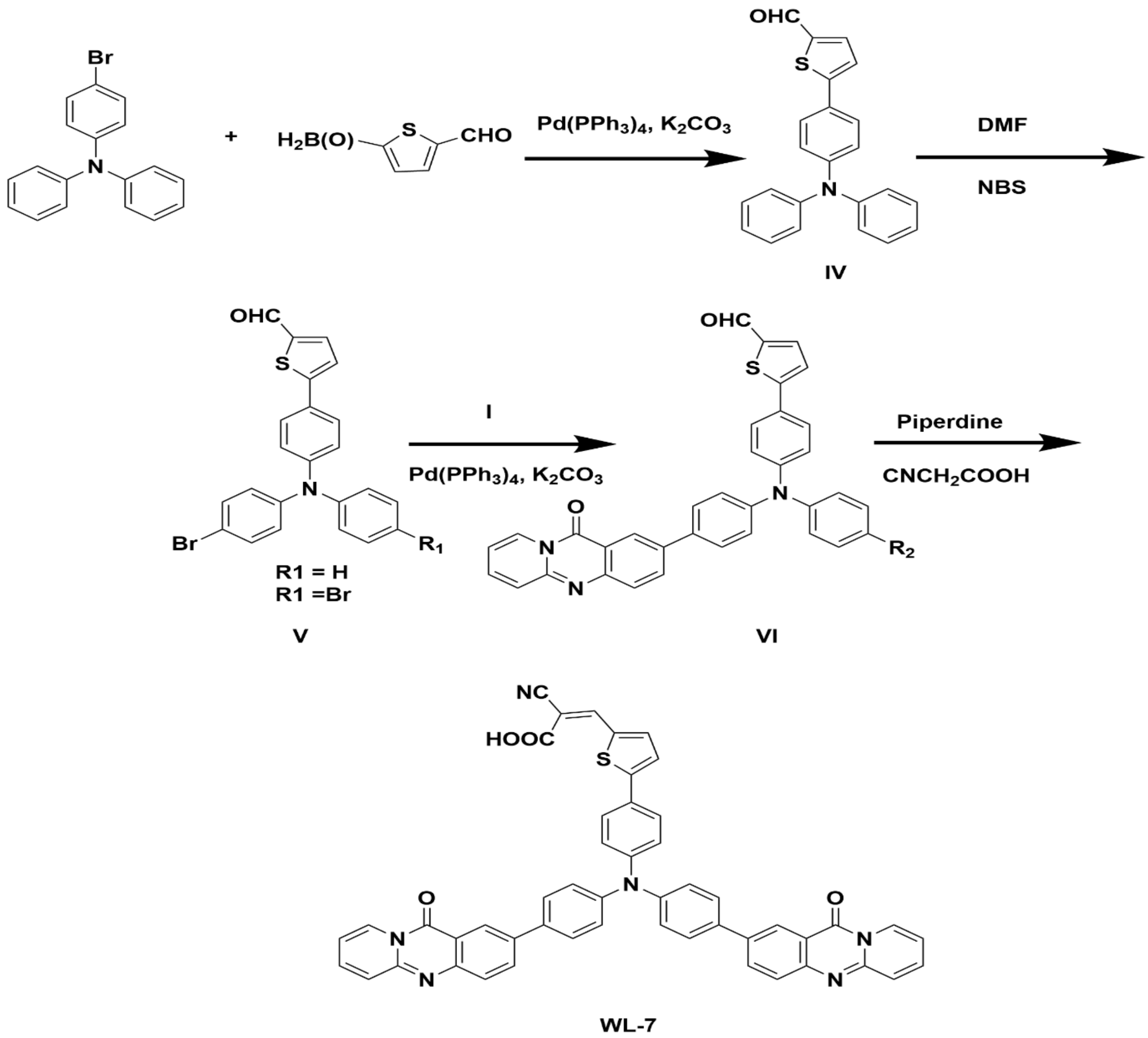

Scheme S1. The synthetic pathway for reference molecule given by Yang Gao et al. [1]

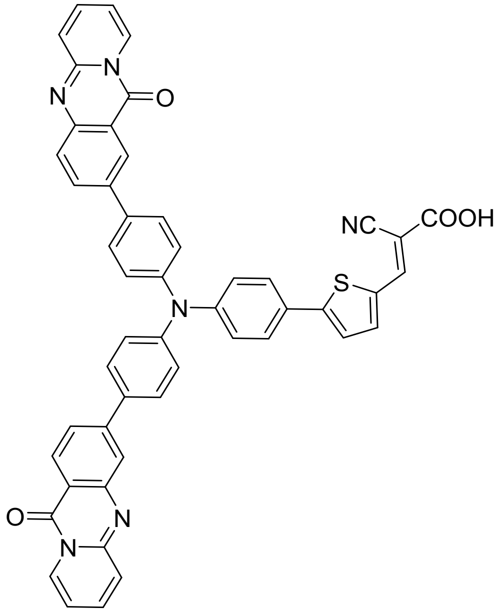

WL-7

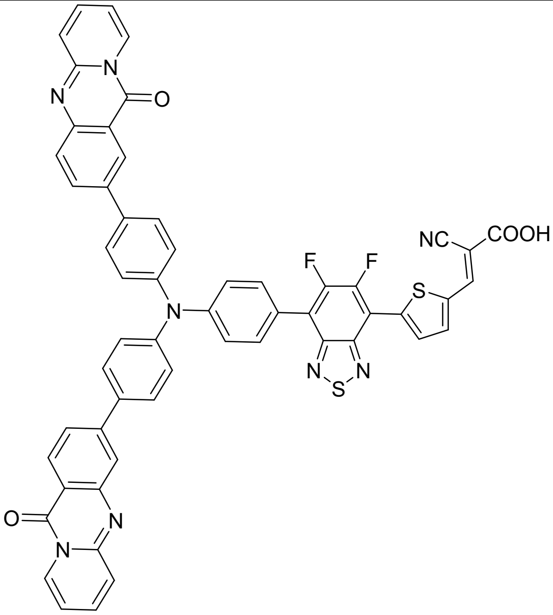

WLK-1

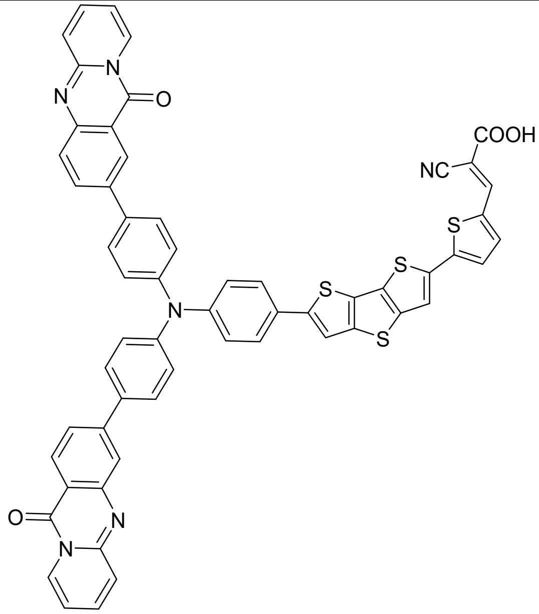

WLK-2

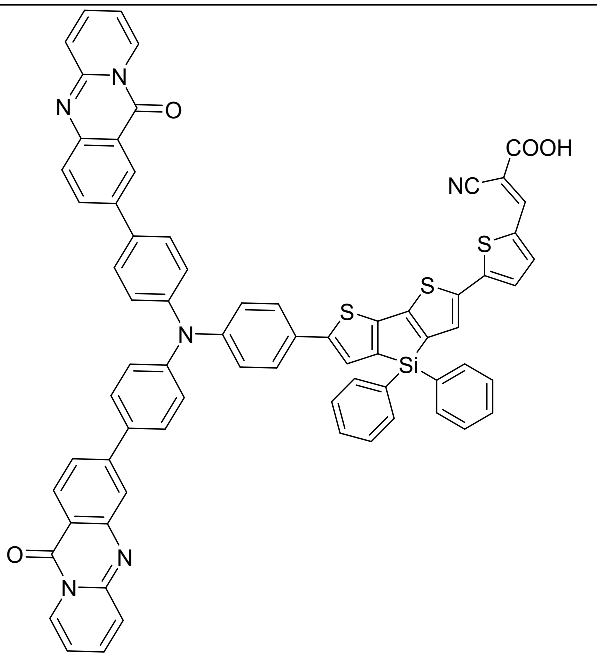

WLK-3

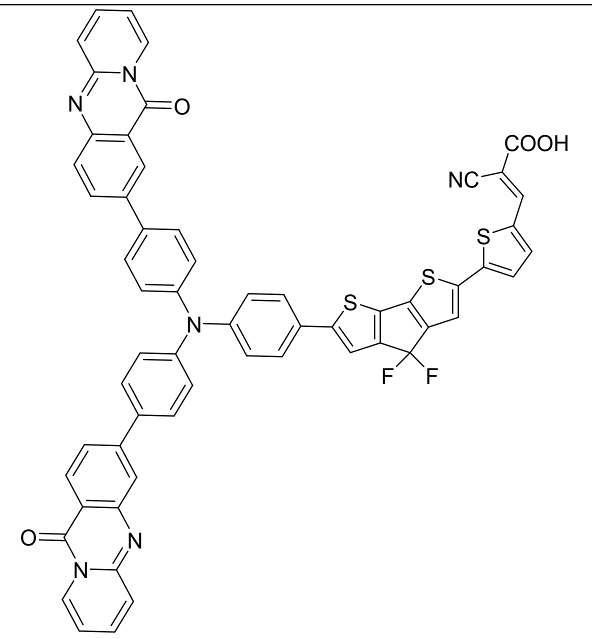

WLK-4

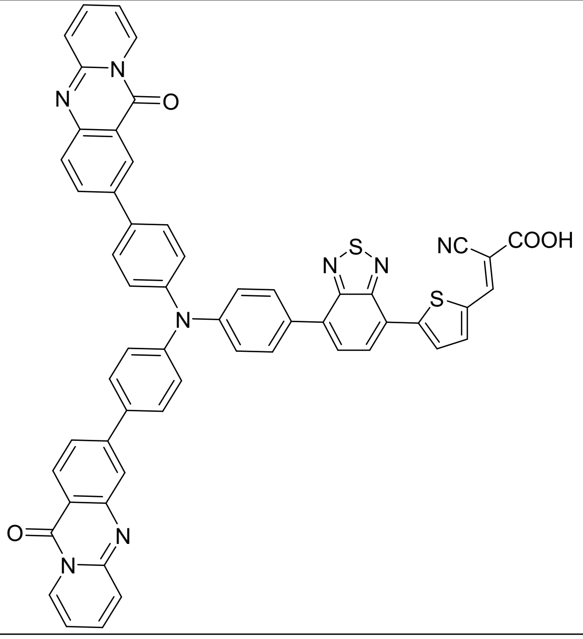

WLK-5

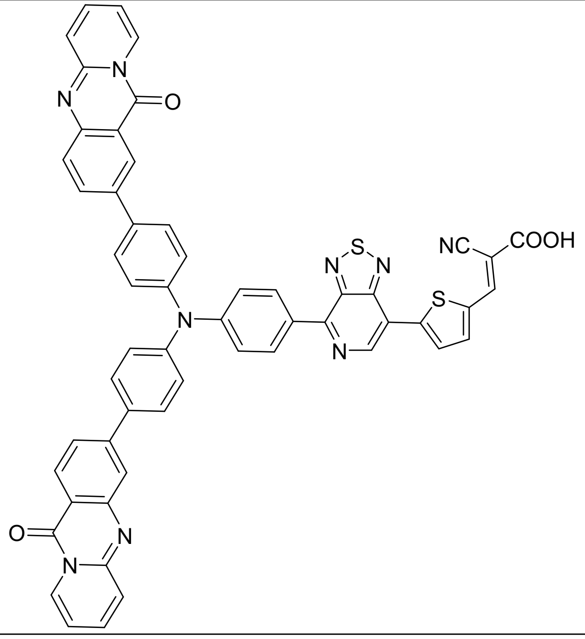

WLK-6

Figure S1. The chemdraw structures of investigated compounds

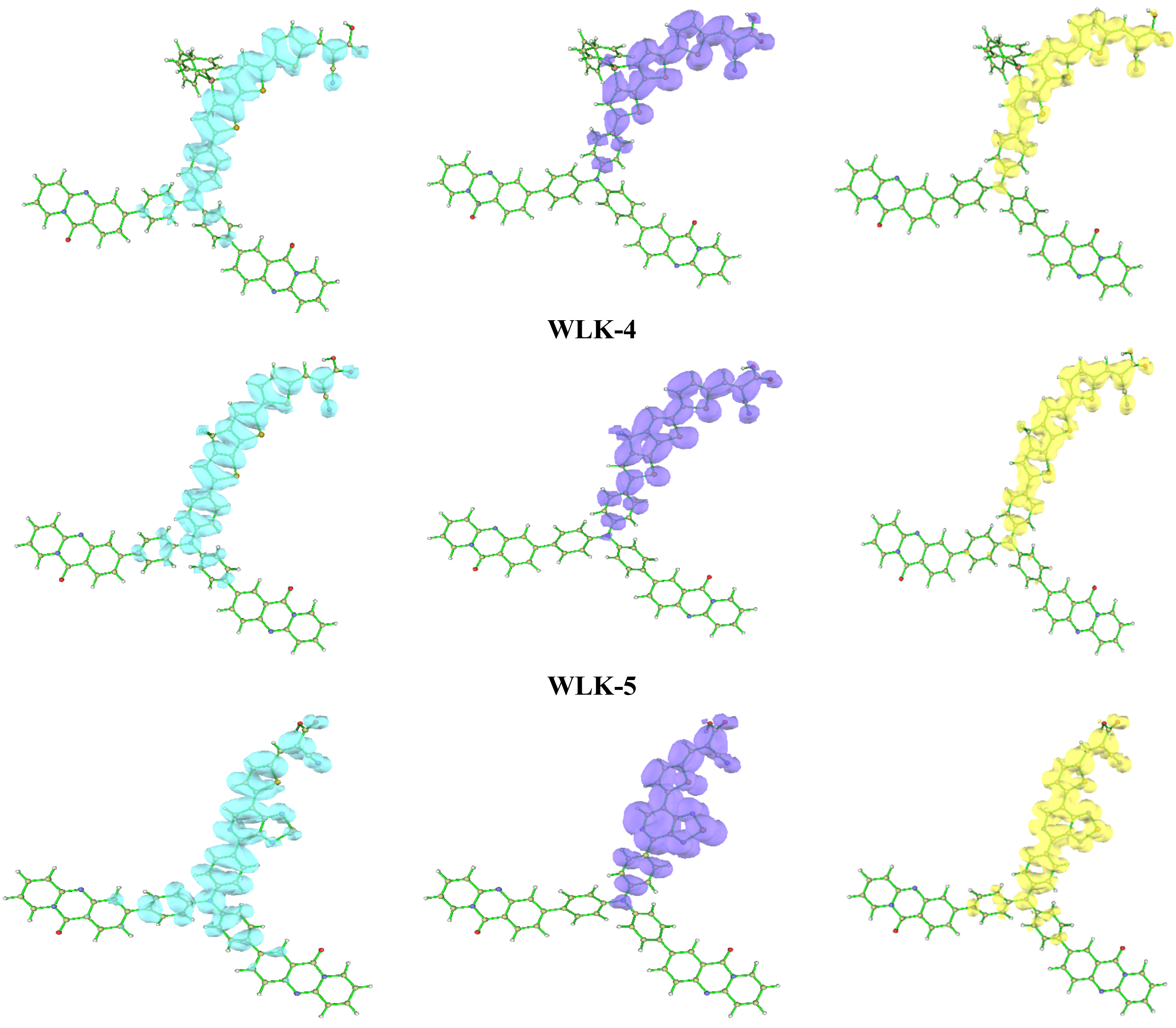

WLK-4

WLK-5

WLK-6

Hole **Electron** **Overlap**

Figure S2. The electron-hole overalap of remaining designed molecules.

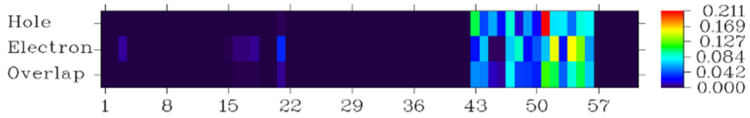

WL-7

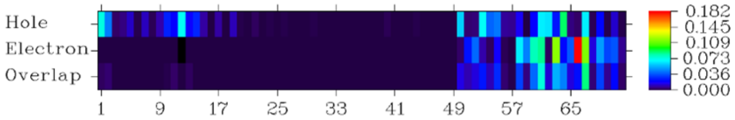

WLK-1

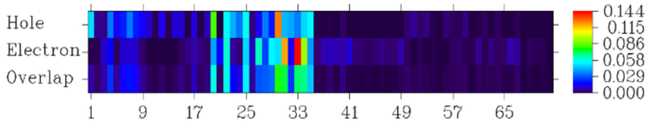

WLK-2

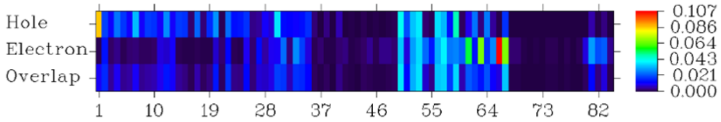

WLK-3

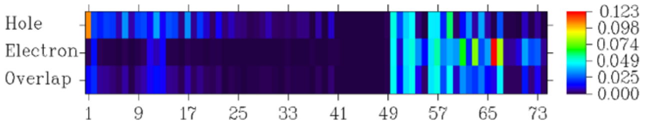

WLK-4

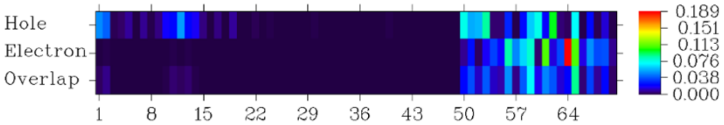

WLK-5

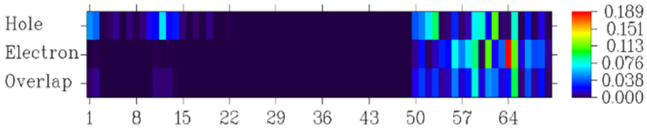

WLK-6

Figure S3. The heat maps of hole electron overlap of all designed chromophores

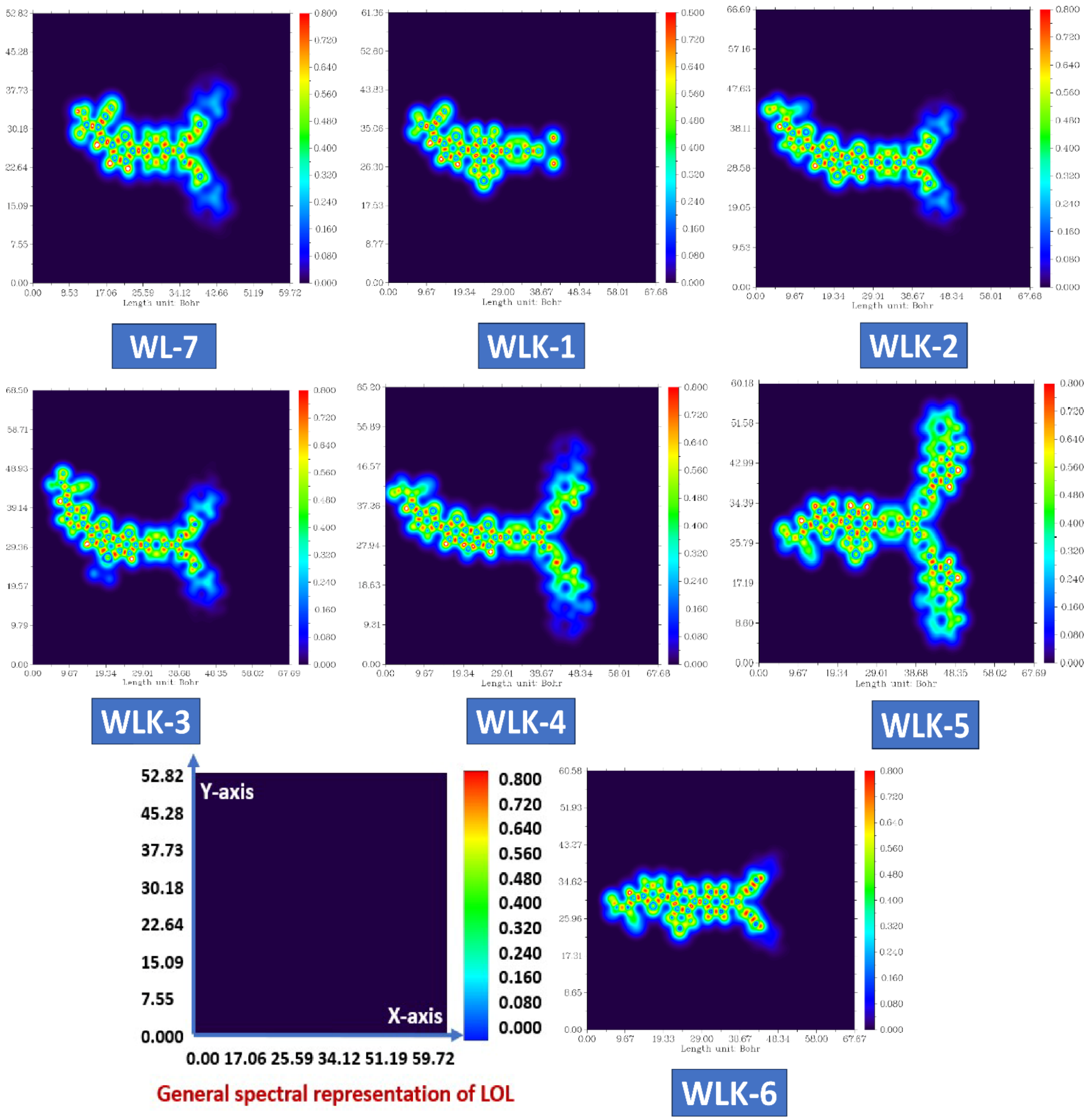

WL-7
WLK-1
WLK-2
WLK-3
WLK-4
WLK-5
Y-axis
X-axis
52.82
45.28
37.73
30.18
22.64
15.09
7.55
0.000
0.00 17.06 25.59 34.12 51.19 59.72
0.800
0.720
0.640
0.560
0.480
0.400
0.320
0.240
0.160
0.080
0.000
Length unit: Bohr
General spectral representation of LOL
WLK-6

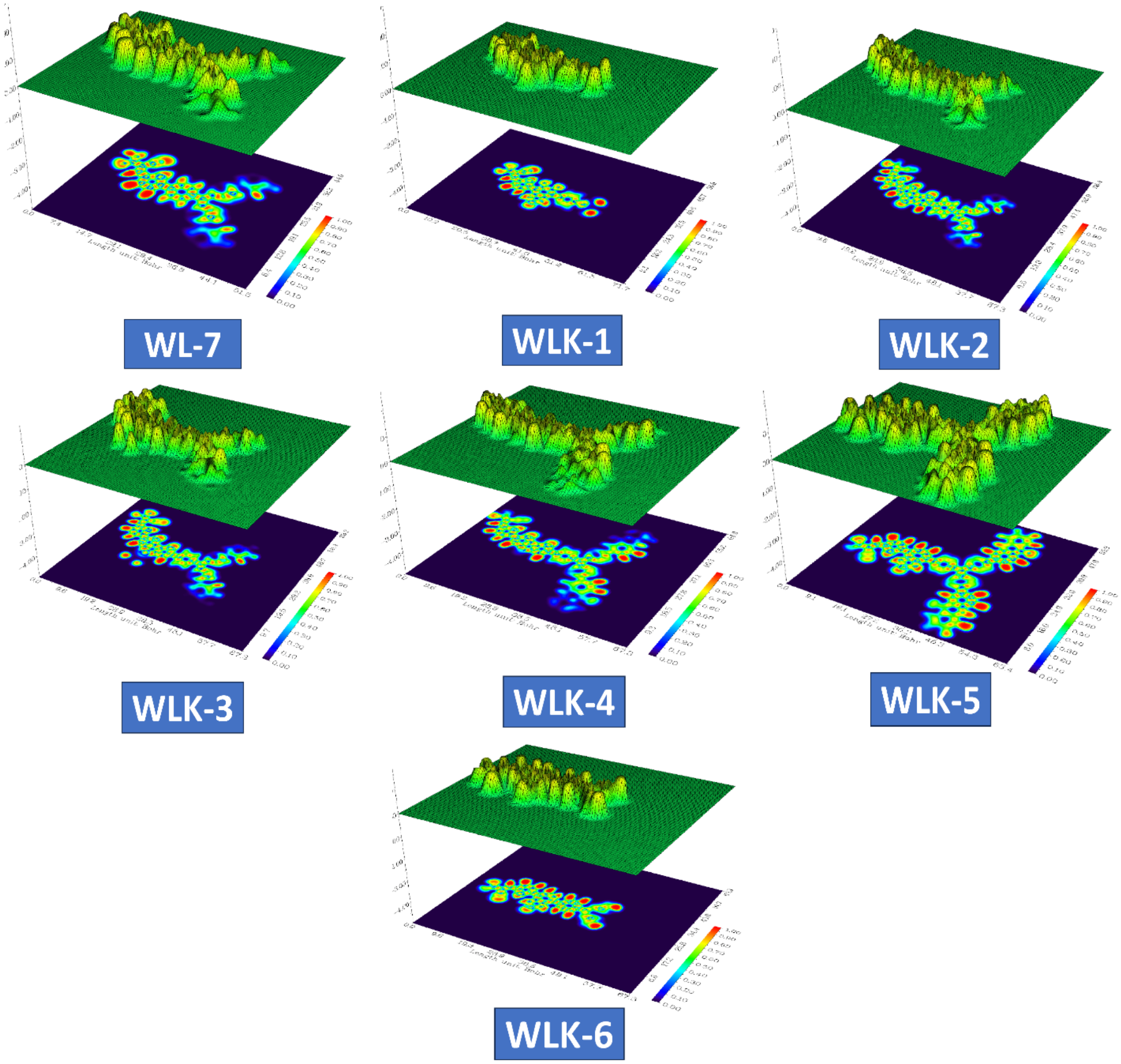

Figure S4. The LOL and ELF plots of all designed compounds.

Table S1: The FMOs, E_{L-H} and rotational velocity of all designed compounds.

Molecules	HOMO (eV)	LUMO (eV)	Energy gap (eV)	Rotational velocity
**WL-7**	-6.349	-1.639	4.710	-8.895
**WLK-1**	-6.428	-2.546	3.882	28.937
**WLK-2**	-6.277	-2.087	4.190	-98.273
**WLK-3**	-6.145	-1.980	4.166	-64.400
**WLK-4**	-6.322	-2.217	4.105	-40.406
**WLK-5**	-6.342	-2.321	4.021	11.444
**WLK-6**	-6.410	-2.558	3.853	25.938

Table S2: The percentage contribution of different fragments in HOMO and LUMO of all designed compounds.

Molecules	HOMO (eV)				LUMO (eV)			
	Donor	Spacer	Additional Spacer	Acceptor	Donor	Spacer	Additional Spacer	Acceptor
**WL-7**	19.8	72.8	0	7.4	50	12.1	0	37.9
**WLK-1**	74.4	4.3	1.3	19.9	4.7	67.1	14.7	13.5
**WLK-2**	68.4	1.8	16	13.8	1.4	34.9	21.1	42.6
**WLK-3**	62.8	23.2	3	11	1.6	25.1	33.3	40
**WLK-4**	68.8	1.8	15.3	14.1	1.9	31.7	30.2	36.1
**WLK-5**	73.8	2	6.6	17.6	5	13.4	58.6	23
**WLK-6**	72.2	3.2	7.8	16.7	9.5	12.4	67.5	10.6

Table S3: Natural bond orbital (NB0) analysis of designed molecules at the DFT level

Molecules	Donor (i)	Type	Acceptor (j)	Type	$E^{(2)}$	Ej.Ej (a.u)	Fi.j (a.u)
**WL-7**	C 2 - C 14	π	C 12 - C 13	π*	35.45	0.36	0.101
	C 3 - C 15	π	C 16 - C 17	π*	31.71	0.37	0.097
	C 4 - C 9	π	C 7 - C 8	π*	30.64	0.37	0.095
	C 7 - C 8	π	C 4 - C 9	π*	32.55	0.34	0.095
	C 16 - C 17	π	C 3 - C 15	π*	31.67	0.34	0.094
	N 1	LP	C 2 - C 14	π*	31.89	0.35	0.098
	N 35	LP	C 38 - C 39	π*	38.67	0.39	0.118

	O 42	LP	C 34 - N 35	σ*	34.56	0.72	0.142
	O 59	LP	C 57 - O 58	σ*	39.21	0.68	0.147
	C 2 - C 10	π	C 11 - C 12	π*	37.34	0.36	0.104
	C 7 - C 8	π	C 4 - C 9	π*	32.5	0.34	0.095
	C 16 - C 17	π	C 3 - C 15	π*	31.72	0.34	0.094
	C 13 - C 14	π	C 2 - C 10	π*	31.35	0.35	0.096
**WLK-1**	C 11 - C 12	π	C 13 - C 14	π*	31.92	0.36	0.097
	N 1	LP	C 2 - C 10	π*	32.79	0.35	0.099
	C 23	LP	C 20 - C 22	π*	71.31	0.21	0.131
	C 23	LP	C 27 - O 35	π*	116.86	0.15	0.139
	O 35	LP	C 27 - N 28	σ*	34.48	0.72	0.142
	C 2 - C 14	π	C 12 - C 13	π*	34.1	0.36	0.099
	C 3 - C 15	π	C 16 - C 17	π*	31.99	0.37	0.097
	C 7 - C 8	π	C 4 - C 9	π*	32.27	0.34	0.095
	C 16 - C 17	π	C 3 - C 15	π*	31.42	0.34	0.093
**WLK-2**	C 23	LP	C 20 - C 22	π*	71.23	0.21	0.131
	O 71	LP	C 70 - O 72	π*	42.53	0.65	0.15
	N 28	LP	C 27 - O 35	π*	57.25	0.35	0.13
	N 28	LP	C 29 - N 30	π*	59.51	0.36	0.133
	N 28	LP	C 31 - C 32	π*	38.64	0.39	0.118
	O 35	LP	C 27 - N 28	σ*	34.5	0.72	0.142
	C 2 - C 14	π	C 12 - C 13	π*	33.22	0.36	0.098
	C 3 - C 15	π	C 16 - C 17	π*	32.32	0.37	0.097
	C 7 - C 8	π	C 4 - C 9	π*	32.08	0.34	0.094
	C 63 - C 64	π	C 66 - C 67	π*	31.79	0.39	0.099
**WLK-3**	C 72 - C 73	π	C 68 - C 74	π*	33.92	0.35	0.097
	C 76 - C 77	π	C 69 - C 75	π*	33.87	0.35	0.097
	C 23	LP	C 20 - C 22	π*	71.13	0.21	0.131
	N 28	LP	C 29 - N 30	π*	59.52	0.36	0.133
	C 38	LP	C 36 - C 37	π*	69.76	0.21	0.131
	N 42	LP	C 43 - N 44	π*	59.69	0.36	0.133
	C 2 - C 14	π	C 12 - C 13	π*	34.51	0.36	0.1
	C 7 - C 8	π	C 4 - C 9	π*	32.35	0.34	0.095
**WLK-4**	C 63 - C 64	π	C 66 - C 67	π*	30.63	0.39	0.098
	C 18 - C 19	π	C 3 - C 15	π*	30.24	0.35	0.094
	C 16 - C 17	π	C 3 - C 15	π*	31.59	0.34	0.094

	N 28	LP	C 27 - O 35	π*	57.28	0.35	0.13
	N 28	LP	C 29 - N 30	π*	59.53	0.36	0.133
	C 38	LP	C 36 - C 37	π*	69.9	0.21	0.131
	N 42	LP	C 43 - N 44	π*	59.76	0.36	0.133
**WLK-5**	C 2 - C 14	π	C 12 - C 13	π*	35.25	0.36	0.101
	C 3 - C 15	π	C 16 - C 17	π*	31.97	0.37	0.097
	C 7 - C 8	π	C 4 - C 9	π*	32.32	0.34	0.095
	C 12 - C 13	π	C 10 - C 11	π*	32.22	0.35	0.096
	C 16 - C 17	π	C 3 - C 15	π*	31.45	0.34	0.093
	C 23	LP	C 20 - C 22	π*	71.21	0.21	0.131
	N 28	LP	C 29 - N 30	π*	59.52	0.36	0.133
	C 38	LP	C 36 - C 37	π*	69.82	0.21	0.131
	N 42	LP	C 43 - N 44	π*	59.79	0.36	0.133
**WLK-6**	O 70	LP	C 68 - O 69	π*	39.59	0.45	0.122
	C 2 - C 14	π	C 12 - C 13	π*	38.08	0.36	0.105
	C 7 - C 8	π	C 4 - C 9	π*	32.73	0.34	0.095
	C 16 - C 17	π	C 3 - C 15	π*	32	0.34	0.094
	C 23 - C 24	π	C 27 - O 35	π*	41	0.32	0.103
	C 38 - C 39	π	C 41 - O 49	π*	42.03	0.32	0.105
	C 52 - C 53	π	C 54 - N 56	π*	32.68	0.34	0.096
	N 28	LP	C 27 - O 35	π*	57.27	0.35	0.13
	N 28	LP	C 29 - N 30	π*	59.55	0.36	0.133
	N 42	LP	C 41 - O 49	π*	56.73	0.35	0.129
	N 42	LP	C 43 - N 44	π*	59.77	0.36	0.133

Table S4: NLO response in a.u at different selected frequencies.

Molecules	NLO properties	Frequencies
	β (0, 0, 0)	
**WL-7**		1.05×10^4
**WLK-1**		2.23×10^4
**WLK-2**		2.16×10^4
**WLK-3**		2.31×10^4

**WLK-4**		2.67 × 10^4
**WLK-5**		2.30 × 10^4
**WLK-6**		3.75 × 10^4
**β (-ω, ω, 0)**	**0.0856 (532 nm)**	**0.0428 (1064 nm)**
**WL-7**	7.30 × 10^4	1.48 × 10^4
**WLK-1**	4.77 × 10^6	3.78 × 10^4
**WLK-2**	2.77 × 10^5	3.26 × 10^4
**WLK-3**	5.85 × 10^5	3.65 × 10^4
**WLK-4**	7.40 × 10^5	4.23 × 10^4
**WLK-5**	1.71 × 10^6	3.78 × 10^4
**WLK-6**	1.41 × 10^7	6.65 × 10^4
**β (-2ω, ω, ω)**	**0.0856 (532 nm)**	**0.0428 (1064 nm)**
**WL-7**	1.87 × 10^5	3.91 × 10^4
**WLK-1**	2.67 × 10^5	5.41 × 10^5
**WLK-2**	8.87 × 10^4	1.17 × 10^5
**WLK-3**	7.00 × 10^5	1.84 × 10^5
**WLK-4**	4.05 × 10^5	2.23 × 10^5
**WLK-5**	9.86 × 10^5	3.24 × 10^5
**WLK-6**	2.33 × 10^6	1.26 × 10^6

Table S5. Photovoltaic characteristics of investigated compounds at the DFT/CAM-B3LYP/6-31G(d,p) level in gas phase.

**Dyes**	**LHE**	E_{ox}^{dye*} **(eV)**	**ΔG^{inject} (eV)**	**ΔG^{regen} (eV)**	**V_{oc} (eV)**
**WL-7**	0.951	3.465	-0.535	1.499	2.361
**WLK-1**	0.891	3.961	-0.039	1.578	1.454
**WLK-2**	0.984	3.556	-0.444	1.427	1.913
**WLK-3**	0.975	3.619	-0.381	1.295	2.020
**WLK-4**	0.978	3.796	-0.204	1.472	1.783
**WLK-5**	0.920	3.917	-0.083	1.492	1.679
**WLK-6**	0.946	4.191	0.191	1.560	1.442

Cartesian Coordinates of optimized geometries

WL-7

N	-0.36939700	-0.01135400	-0.07677300
C	-0.00173700	1.35178200	-0.13421400
C	-1.74166400	-0.39185800	-0.07405800
C	0.63182100	-1.02635300	-0.01982700
C	1.74266100	-0.98266400	-0.86891800
C	2.71015700	-1.97721600	-0.81035200
C	2.59666800	-3.05140500	0.08360700
C	1.47486200	-3.08718300	0.92345700
C	0.50950800	-2.08942700	0.88023700
C	-0.77451400	2.27347200	-0.85793100
C	-0.41312300	3.60950700	-0.91002700
C	0.73256800	4.08242800	-0.24880700
C	1.50595600	3.15148100	0.46202500
C	1.14699100	1.81445200	0.52456700
C	-2.17734500	-1.47384300	-0.84674800
C	-3.51215600	-1.85671800	-0.83010900
C	-4.45705300	-1.16683200	-0.05844700
C	-4.00987300	-0.08197900	0.70840200
C	-2.67423200	0.29713500	0.71019600
C	3.62964800	-4.11166100	0.13922600
C	-5.88301700	-1.56972400	-0.05369000
C	1.09196600	5.49363700	-0.30899100
C	0.29293900	6.56577500	-0.62923300
C	0.95869800	7.81128900	-0.59609000
C	2.29000900	7.74566300	-0.24523400
S	2.76539300	6.03048700	0.05481400

C	3.14807700	8.87585800	-0.14004000
C	4.46103100	8.97220100	0.20386500
C	4.97007200	-3.82135400	-0.06263100
C	5.94211200	-4.83255600	-0.01052300
C	5.57567200	-6.16747200	0.25500700
C	4.21281900	-6.45956200	0.46001900
C	3.27018200	-5.45878400	0.40134700
C	7.33210000	-4.49638900	-0.22347400
N	8.23231700	-5.60362800	-0.13936900
C	7.76776700	-6.90638600	0.12839900
N	6.50014300	-7.18845700	0.31821100
C	9.58507200	-5.34039200	-0.33392400
C	10.50322900	-6.32973100	-0.27307900
C	10.07778800	-7.67138100	-0.00425200
C	8.76186700	-7.94005700	0.18735000
O	7.77582000	-3.36060100	-0.46506300
C	-6.24026000	-2.93989000	-0.14382700
C	-7.56162500	-3.32439100	-0.14040800
C	-8.57946400	-2.35921000	-0.04461500
C	-8.24412700	-0.99298800	0.04905500
C	-6.88874400	-0.61957900	0.04002200
C	-9.96352400	-2.76780100	-0.03998100
N	-10.90093600	-1.69139600	0.06067200
C	-10.47040000	-0.35298200	0.14607400
N	-9.20526000	-0.00559600	0.14122500
C	-12.25211800	-2.02258200	0.06936400
C	-13.20355100	-1.06743500	0.16025200
C	-12.81413800	0.30867500	0.24889200

C	-11.49960300	0.64371800	0.24140900
O	-10.37859700	-3.93760000	-0.11437000
C	5.05176100	10.31939600	0.21205200
O	6.36723700	10.31579800	0.56891500
O	4.44967600	11.35954600	-0.06826900
C	5.28047100	7.86281000	0.55203700
N	5.94150800	6.94333300	0.83684600
H	1.83946700	-0.17219000	-1.58121300
H	3.55080600	-1.93730200	-1.49347000
H	1.37257300	-3.88730100	1.64776000
H	-0.34032900	-2.12463700	1.55111300
H	-1.65210700	1.93207400	-1.39174500
H	-1.01225100	4.29171600	-1.50157200
H	2.38814100	3.48173500	0.99892800
H	1.75229800	1.12172900	1.09480900
H	-1.46608600	-2.00701100	-1.46557800
H	-3.83119800	-2.68274600	-1.45534900
H	-4.71154400	0.44934400	1.34108400
H	-2.34519400	1.12297400	1.32909400
H	-0.75861500	6.46523700	-0.85870300
H	0.47272100	8.75409700	-0.81487000
H	2.67626900	9.82822500	-0.37431200
H	5.30558400	-2.80710000	-0.24283900
H	3.94204100	-7.48975300	0.65224900
H	2.22338300	-5.70634000	0.53630000
H	9.80083700	-4.30058800	-0.52966900
H	11.54888900	-6.10200900	-0.42708400
H	10.81236700	-8.46589000	0.04469800

H	8.38636500	-8.93237100	0.39332300
H	-5.46034500	-3.69017700	-0.18879100
H	-7.84744400	-4.36751200	-0.20054400
H	-6.66704400	0.43891200	0.08366900
H	-12.43856200	-3.08383300	-0.00194500
H	-14.24773400	-1.34781300	0.16533300
H	-13.57495100	1.07626900	0.32162700
H	-11.15087400	1.66466700	0.30574900
H	6.73131800	11.22340800	0.56493100

WLK-1

N	-2.08083400	0.24992400	0.05020700
C	-0.69500900	0.00327400	0.16766000
C	-3.00922900	-0.82944300	0.00238900
C	-2.57161900	1.58780100	-0.02179400
C	-2.10426800	2.56784000	0.86020000
C	-2.58868700	3.86715500	0.78602900
C	-3.56122700	4.22849200	-0.15713500
C	-4.02839700	3.23462200	-1.02832300
C	-3.53928900	1.93583100	-0.96910800
C	-0.21932300	-1.09809300	0.89422000
C	1.14096000	-1.33630400	1.01048000
C	2.08229700	-0.48785600	0.40572600
C	1.59804100	0.61453500	-0.31888400
C	0.23924100	0.85794600	-0.43587900
C	-4.20201300	-0.76869800	0.73074200
C	-5.11670200	-1.81190900	0.66887200

C	-4.86673400	-2.95294700	-0.10550300
C	-3.66657500	-3.00424100	-0.82801500
C	-2.75430300	-1.95852400	-0.78448200
C	-4.07442100	5.61607300	-0.23106500
C	-5.83866400	-4.07029900	-0.15832000
C	-3.24894900	6.70165000	0.01732800
C	-3.74287300	8.01361800	-0.05249300
C	-5.09141800	8.25590700	-0.38353400
C	-5.92801900	7.15080900	-0.63597200
C	-5.43104200	5.86989800	-0.55948500
C	-2.85520100	9.12417400	0.20977200
N	-3.46504300	10.41228500	0.10250400
C	-4.82654200	10.55239800	-0.23174100
N	-5.61411700	9.52903100	-0.46583900
C	-2.65837500	11.52085600	0.34268500
C	-3.15558700	12.77463700	0.26389500
C	-4.53532700	12.96667700	-0.07204600
C	-5.33130500	11.89385500	-0.30855800
O	-1.65177100	9.03681400	0.50997000
C	-7.23268800	-3.80936400	-0.11688100
C	-8.14338800	-4.83999400	-0.16469900
C	-7.70138400	-6.17138400	-0.25836500
C	-6.32014100	-6.45036800	-0.30432700
C	-5.40515000	-5.38431400	-0.25009000
C	-8.66049400	-7.24846600	-0.30966300
N	-8.08348300	-8.55448200	-0.40361600
C	-6.68708200	-8.73557400	-0.43997200
N	-5.83401200	-7.73994800	-0.39273200

C	-8.95845400	-9.63480800	-0.45641600
C	-8.49734300	-10.90183500	-0.54470100
C	-7.08428700	-11.13615800	-0.58434700
C	-6.22139800	-10.09062300	-0.53352400
O	-9.89748700	-7.12523600	-0.27888900
C	3.52813300	-0.74348400	0.51625900
C	4.14170200	-1.30359700	1.70447300
C	5.58901700	-1.53380300	1.79602000
C	6.47203800	-1.21391800	0.68899400
C	5.82202900	-0.69366500	-0.39345000
C	4.41705300	-0.47006500	-0.47906400
F	3.99787700	0.04304700	-1.68479100
F	6.52226600	-0.34851000	-1.52222800
N	3.45975200	-1.62936100	2.78622400
S	4.58961300	-2.24407700	3.97768700
N	5.99672000	-2.03616100	2.94570600
C	7.90809900	-1.43077600	0.74513200
C	8.63427100	-1.94125300	1.79710100
C	10.02068200	-2.03279100	1.54711800
C	10.41065000	-1.59713100	0.30121900
S	8.99209600	-1.03033300	-0.64314300
C	11.76524500	-1.61076900	-0.16058200
C	12.30954300	-1.19278900	-1.33261500
C	11.55921800	-0.57945200	-2.37636400
N	10.95177600	-0.08320800	-3.24055700
C	13.78097200	-1.26482300	-1.56285500
O	14.39169700	-0.49753600	-2.28745500
O	14.45929700	-2.25198300	-0.87814600

H	-1.36775600	2.30417400	1.60964700
H	-2.23114300	4.60595000	1.49409100
H	-4.75821000	3.48799400	-1.78889500
H	-3.89815700	1.18641400	-1.66418100
H	-0.92215900	-1.76495400	1.37655700
H	1.47685800	-2.17956100	1.59643600
H	2.29159200	1.28795100	-0.80313500
H	-0.10463500	1.71302200	-1.00314300
H	-4.40382100	0.09594400	1.35109500
H	-6.02299300	-1.75399700	1.26073000
H	-3.46156700	-3.85916200	-1.46201100
H	-1.84384000	-2.00740100	-1.36926500
H	-2.19898200	6.56890200	0.24888300
H	-6.96409200	7.34873200	-0.87872700
H	-6.09547100	5.03085600	-0.73217100
H	-1.63721600	11.26931100	0.58749800
H	-2.51033100	13.62106600	0.45415000
H	-4.93571200	13.97110400	-0.13555000
H	-6.37742700	11.98086800	-0.56578800
H	-7.58007200	-2.78432500	-0.07397600
H	-9.21025500	-4.65375400	-0.14187900
H	-4.35234700	-5.63531800	-0.25759100
H	-9.99976100	-9.35116500	-0.42008700
H	-9.19566300	-11.72628100	-0.58467800
H	-6.71213200	-12.15096500	-0.65502300
H	-5.14759700	-10.21035900	-0.56007400
H	8.16485400	-2.23812500	2.72115200
H	10.72782800	-2.41253100	2.27421100

H	12.45935400	-1.99829100	0.58223800
H	13.89530400	-2.99060400	-0.58354200

WLK-2

N	3.07519200	0.40613600	-0.07927000
C	1.78971000	-0.18124000	-0.16931900
C	4.23913200	-0.41076700	-0.03299000
C	3.21255300	1.82434300	-0.03303300
C	2.47452900	2.64136100	-0.89645200
C	2.61163500	4.02223200	-0.84517500
C	3.49524700	4.63331700	0.05570100
C	4.23542600	3.80337300	0.90913100
C	4.09418500	2.42204400	0.87350000
C	1.58420800	-1.34307300	-0.92769700
C	0.32460700	-1.91477000	-1.01483700
C	-0.77816500	-1.35249900	-0.35201100
C	-0.56561600	-0.18436000	0.39473200
C	0.69302600	0.39015700	0.49020200
C	5.38392800	-0.05620200	-0.75581000
C	6.52450300	-0.84617600	-0.69807800
C	6.55825800	-2.02099900	0.06543900
C	5.40448300	-2.36891100	0.78141300
C	4.26540500	-1.57620700	0.74248500
C	3.63785500	6.10681600	0.10526100
C	7.77240600	-2.86885200	0.11314800
C	2.55278300	6.94182400	-0.11186200
C	2.69638800	8.33729200	-0.06536900
C	3.94956100	8.92093600	0.20984900

C	5.05048200	8.07009100	0.43068500
C	4.89574400	6.70375600	0.37749100
C	1.54421700	9.18024500	-0.29365500
N	1.80737300	10.58287400	-0.21469800
C	3.09945200	11.07106200	0.06323500
N	4.13139700	10.28647800	0.26763500
C	0.73485000	11.44482000	-0.42453000
C	0.89680100	12.78502200	-0.36940100
C	2.19325600	13.32812900	-0.09068400
C	3.24615100	12.49794700	0.11585600
O	0.39258800	8.78406500	-0.54390600
C	9.06135500	-2.27666200	0.07820000
C	10.19527700	-3.05522600	0.12124000
C	10.09028300	-4.45487500	0.20351700
C	8.81818800	-5.06159000	0.24294300
C	7.67157300	-4.24966800	0.19373100
C	11.28238500	-5.26673400	0.24962200
N	11.04016700	-6.67466500	0.33122000
C	9.72963700	-7.19013100	0.36172000
N	8.66016300	-6.43148800	0.31980900
C	12.15149200	-7.51023800	0.37788900
C	12.01227900	-8.85208200	0.45440300
C	10.69855600	-9.42329000	0.48783000
C	9.60739900	-8.61854800	0.44288800
O	12.45241400	-4.84629700	0.22404600
C	-2.09370500	-1.97761300	-0.44557100
C	-2.40070400	-3.28428000	-0.70689900
C	-3.79958800	-3.53073100	-0.73134900

C	-4.59370400	-2.43057100	-0.48471300
S	-3.59821400	-0.97389000	-0.21337200
S	-4.77368200	-5.02812700	-1.00622200
C	-6.26917200	-4.04151500	-0.76554900
C	-5.97861900	-2.71657500	-0.50294600
C	-7.64823800	-4.36074300	-0.78280800
C	-8.44911500	-3.27424500	-0.53977000
S	-7.46703800	-1.76477400	-0.26581000
C	-9.88574900	-3.24068800	-0.49957200
C	-10.76082400	-4.16680600	-1.02004200
C	-12.11938400	-3.85383600	-0.79824600
C	-12.33806700	-2.67824400	-0.11268600
S	-10.76562900	-1.90408400	0.31287600
C	-13.63076700	-2.15774500	0.19804900
C	-14.00065800	-1.04136400	0.88000700
C	-13.07114900	-0.14853600	1.48537400
N	-12.31134200	0.58949300	1.97583100
C	-15.43708600	-0.73198000	1.12394100
O	-15.84219100	-0.10382100	2.08756500
O	-16.33447900	-1.22543800	0.19811000
H	1.80077600	2.18893800	-1.61399300
H	2.04813900	4.63522400	-1.53927700
H	4.90472500	4.24499800	1.63879400
H	4.66003800	1.79955300	1.55597100
H	2.41526100	-1.78575800	-1.46203700
H	0.18583400	-2.79259800	-1.63528600
H	-1.39060200	0.26640100	0.93507000
H	0.83382900	1.28240300	1.08685100

H	5.37157700	0.83640000	-1.36910700
H	7.39057500	-0.56415300	-1.28586500
H	5.41158700	-3.25397100	1.40719300
H	3.39315600	-1.85111500	1.32281700
H	1.56373800	6.54181200	-0.30016900
H	6.00987900	8.52966600	0.63049300
H	5.75919300	6.06497800	0.52483200
H	-0.19646300	10.93709400	-0.62671200
H	0.04927200	13.43553200	-0.53530100
H	2.32503300	14.40232200	-0.04624600
H	4.24423100	12.85315600	0.32982100
H	9.14910700	-1.19761200	0.04404200
H	11.18483500	-2.61492200	0.10320600
H	6.71141600	-4.74920400	0.19579600
H	13.09258800	-6.98157200	0.34701200
H	12.89008400	-9.48229800	0.48972300
H	10.58426800	-10.49871100	0.54905700
H	8.59491800	-8.99601700	0.46510100
H	-1.64821300	-4.04737600	-0.84694300
H	-8.04109400	-5.35511100	-0.94406900
H	-10.43218300	-5.03771200	-1.56980700
H	-12.93577200	-4.47496600	-1.14586500
H	-14.43787700	-2.79672400	-0.15412300
H	-15.93952600	-1.42168600	-0.67133000

WLK-3

N	3.74738200	0.93697100	-0.03022500
C	2.43052300	0.41067800	0.00229000

C	4.86829900	0.06356800	-0.06771600
C	3.95181600	2.34696000	-0.02529800
C	3.20928400	3.17254700	0.82634900
C	3.41014300	4.54650100	0.82430400
C	4.36326100	5.14221200	-0.01376400
C	5.10618000	4.30381300	-0.85647500
C	4.90195200	2.93008300	-0.87054900
C	2.13808300	-0.74050600	0.74685700
C	0.84997500	-1.25217300	0.77769800
C	-0.19601100	-0.63707900	0.07060900
C	0.10465500	0.52102300	-0.66184800
C	1.39257900	1.03484400	-0.70148000
C	6.01507200	0.34495300	0.68440300
C	7.11366200	-0.50280700	0.63598300
C	7.10259600	-1.66532000	-0.14687500
C	5.94720200	-1.94039700	-0.89134000
C	4.85049700	-1.08986500	-0.86174300
C	4.57415600	6.60827400	-0.01052600
C	8.27168700	-2.57443800	-0.18510200
C	3.51869800	7.48633100	0.18179400
C	3.72723700	8.87434300	0.18593000
C	5.01717200	9.40689600	-0.01218000
C	6.08784700	8.51255000	-0.20801800
C	5.86924300	7.15388000	-0.20484900
C	2.60460300	9.76298900	0.38667900
N	2.93425100	11.15365700	0.36414900
C	4.25885600	11.58912500	0.16205600
N	5.26297900	10.76374900	-0.01861700

C	1.89279500	12.05794500	0.55082000
C	2.11730600	13.39023500	0.54533500
C	3.44877600	13.88041700	0.34437600
C	4.47192700	13.00863700	0.16109800
O	1.42563900	9.41382300	0.57032700
C	9.58864000	-2.05127800	-0.11131500
C	10.68148500	-2.88688400	-0.14538500
C	10.50623400	-4.27743600	-0.25728500
C	9.20552200	-4.81600600	-0.33559900
C	8.10168200	-3.94659700	-0.29474300
C	11.65542800	-5.14921000	-0.29402400
N	11.34264600	-6.54093900	-0.40819200
C	10.00814700	-6.98671400	-0.47660300
N	8.97872900	-6.17439600	-0.44265600
C	12.41001400	-7.43225800	-0.44754400
C	12.20347800	-8.76340200	-0.55320900
C	10.86301400	-9.26470900	-0.62569600
C	9.81420000	-8.40520900	-0.58824100
O	12.84490400	-4.79106400	-0.23558200
C	-1.54234000	-1.20066500	0.10660300
C	-1.92975600	-2.48985900	0.36773100
C	-3.33703200	-2.70678900	0.33217800
C	-4.04539000	-1.56314200	0.03500600
S	-2.98758900	-0.15036500	-0.20724300
Si	-4.58657500	-4.13369000	0.56847200
C	-6.02610000	-2.92639000	0.20936800
C	-5.48170200	-1.68034600	-0.03395800
C	-7.44136700	-2.93211300	0.11517900

C	-7.99935500	-1.71394200	-0.19923400
S	-6.71382600	-0.45051100	-0.39645300
C	-9.39009500	-1.40281400	-0.37292600
C	-10.44010800	-2.28762300	-0.49320700
C	-11.69677300	-1.66475600	-0.63795600
C	-11.66503400	-0.28578300	-0.64360800
S	-9.96794100	0.29517900	-0.44713000
C	-12.81643500	0.54368900	-0.78065200
C	-12.93994500	1.89762800	-0.83642300
C	-4.67741200	-4.84498100	2.32097800
C	-4.35789400	-5.50420600	-0.71769300
C	-3.92302800	-5.97232400	2.67956700
C	-3.95530300	-6.46829300	3.98168200
C	-4.74503900	-5.84441800	4.94603300
C	-5.50193600	-4.72460700	4.60523400
C	-5.46777300	-4.22963600	3.30303600
C	-3.59053500	-5.28195900	-1.87065100
C	-3.45466200	-6.27443200	-2.83960600
C	-4.08499600	-7.50595300	-2.67027900
C	-4.84977700	-7.74350000	-1.52923900
C	-4.98359800	-6.75035700	-0.56076600
C	-11.82874000	2.78715400	-0.80544100
N	-10.92059100	3.51988400	-0.77074500
C	-14.26787700	2.53939200	-1.03468200
O	-14.42762600	3.62169200	-1.57429800
O	-15.36459600	1.82569900	-0.59117200
H	2.48119600	2.73153800	1.49623800
H	2.84070000	5.16436500	1.50920800

H	5.82903700	4.73490600	-1.53994200
H	5.47184900	2.30275600	-1.54512900
H	2.92410100	-1.22294400	1.31427400
H	0.64310800	-2.12375000	1.38764900
H	-0.67471400	1.01289100	-1.23310000
H	1.60170300	1.92119600	-1.28709900
H	6.03680000	1.22631400	1.31339300
H	7.98034100	-0.27621600	1.24649100
H	5.92084100	-2.81451300	-1.53188400
H	3.97730200	-1.30977500	-1.46355000
H	2.50472100	7.12742600	0.31109200
H	7.07551100	8.93258200	-0.34835200
H	6.70908100	6.48014900	-0.33156500
H	0.93095600	11.58829400	0.69345800
H	1.29291200	14.07423500	0.69199000
H	3.63074900	14.94818100	0.33984900
H	5.49409000	13.32322300	0.00506400
H	9.73131300	-0.97909100	-0.05433600
H	11.69180700	-2.49906200	-0.09773600
H	7.11724600	-4.39522500	-0.32671600
H	13.37622100	-6.95391000	-0.38633500
H	13.04813500	-9.43770600	-0.58213800
H	10.69472400	-10.33142900	-0.71028400
H	8.78429500	-8.72893600	-0.63925600
H	-1.20958800	-3.27476600	0.55939600
H	-8.04894000	-3.81158800	0.28722100
H	-10.30385100	-3.35990800	-0.49551000
H	-12.62259700	-2.21600300	-0.74929000

H	-13.73631600	-0.02783300	-0.88601500
H	-3.31080800	-6.47375200	1.93654300
H	-3.36642800	-7.34072500	4.24248400
H	-4.77141300	-6.23020700	5.95912300
H	-6.11841900	-4.23727200	5.35238600
H	-6.06239900	-3.35832500	3.04842900
H	-3.09265100	-4.32807300	-2.01096800
H	-2.85661400	-6.08696800	-3.72443600
H	-3.97886400	-8.27875000	-3.42354400
H	-5.33896000	-8.70161500	-1.39243400
H	-5.57269000	-6.95329700	0.32828700
H	-15.15355700	1.15977200	0.08878200

WLK-4

N	-3.18079000	0.41304300	-0.00276300
C	-1.85674500	-0.08547600	-0.02531300
C	-4.28893500	-0.48038800	-0.01277200
C	-3.41769100	1.81873400	0.03180600
C	-2.69923300	2.68450400	-0.79993900
C	-2.93441000	4.05250200	-0.76034000
C	-3.89941000	4.60087200	0.09633500
C	-4.61913800	3.72188100	0.91750600
C	-4.38034100	2.35373800	0.89385100
C	-1.53769900	-1.24174700	-0.75287600
C	-0.23960200	-1.72652800	-0.77403900
C	0.79109900	-1.07983000	-0.07286800
C	0.46509400	0.08088400	0.64432900

C	-0.83228000	0.56910300	0.67298200
C	-5.41135700	-0.20819600	-0.80282900
C	-6.49915400	-1.07141100	-0.79946200
C	-6.49876200	-2.23930200	-0.02467500
C	-5.36713000	-2.50428600	0.75891700
C	-4.28216300	-1.63814000	0.77419600
C	-4.14625400	6.06090800	0.13343800
C	-7.65568700	-3.16506400	-0.03436900
C	-3.11226700	6.96897400	-0.03367900
C	-3.35429400	8.35113100	0.00002700
C	-4.65696500	8.84655900	0.21081600
C	-5.70580000	7.92148200	0.38130700
C	-5.45433700	6.56914500	0.34159800
C	-2.25340100	9.27180800	-0.17471300
N	-2.61694300	10.65280200	-0.11661600
C	-3.95188300	11.05035500	0.09529100
N	-4.93566400	10.19615800	0.25325700
C	-1.59792900	11.58695000	-0.27886100
C	-1.85517200	12.91271600	-0.23906000
C	-3.19827700	13.36471700	-0.02660000
C	-4.19978100	12.46360600	0.13306600
O	-1.06588700	8.95614600	-0.36457800
C	-8.97658800	-2.65922500	-0.14635500
C	-10.05780000	-3.51051700	-0.15697000
C	-9.86612400	-4.89954200	-0.05388400
C	-8.56126700	-5.42089200	0.06167800
C	-7.46918800	-4.53549800	0.06665300
C	-11.00333600	-5.78790100	-0.06428100

N	-10.67442100	-7.17615100	0.04521500
C	-9.33670200	-7.60392300	0.15296200
N	-8.31846600	-6.77668400	0.16184300
C	-11.72957500	-8.08284400	0.03959100
C	-11.50723200	-9.41202700	0.13767400
C	-10.16282900	-9.89513700	0.24905300
C	-9.12585800	-9.02052800	0.25563800
O	-12.19511000	-5.44549800	-0.15797900
C	2.14709200	-1.61601700	-0.09920100
C	2.56141700	-2.90487900	-0.32658300
C	3.96726700	-3.04648800	-0.28936400
C	4.65556700	-1.88898300	-0.03033400
S	3.57114800	-0.51099900	0.17519700
C	4.95254400	-4.18205000	-0.44163400
C	6.28781400	-3.50664300	-0.23368500
C	6.07550200	-2.17004600	0.00520900
C	7.64146500	-3.89650600	-0.23980800
C	8.49944300	-2.84879500	0.00675200
S	7.58322200	-1.29271400	0.25326900
C	9.93313800	-2.88524100	0.09046600
C	10.73540000	-3.99603700	0.22287000
C	12.11518600	-3.70028900	0.25980700
C	12.42346200	-2.36017000	0.16730800
S	10.91466700	-1.38520900	0.00299500
C	13.75022200	-1.83435200	0.20712000
C	14.20413700	-0.55679500	0.10730200
F	4.72694700	-5.21125200	0.48735800
F	4.86216200	-4.80461400	-1.69704700

C	13.35151000	0.56232800	-0.11229100
N	12.65281300	1.48159400	-0.28362000
C	15.66294900	-0.25627200	0.12471500
O	16.16450800	0.70319200	-0.43659400
O	16.46422800	-1.15327900	0.80197500
H	-1.96352400	2.27969900	-1.48434400
H	-2.38370000	4.70297900	-1.43015000
H	-5.35132500	4.11620700	1.61306300
H	-4.93297600	1.69345900	1.55119500
H	-2.31091600	-1.74878900	-1.31588600
H	-0.01359300	-2.60187000	-1.37188000
H	1.23095200	0.59475400	1.21482400
H	-1.06048300	1.45743400	1.24790300
H	-5.42267600	0.67827700	-1.42501800
H	-7.34730800	-0.85240700	-1.43799600
H	-5.35159400	-3.38276800	1.39370800
H	-3.42789900	-1.84976900	1.40562400
H	-2.08987100	6.63841200	-0.17201200
H	-6.70340200	8.31352700	0.53181800
H	-6.27760500	5.87203500	0.44920200
H	-0.62483000	11.14498500	-0.43295200
H	-1.04788000	13.62036500	-0.36751700
H	-3.40643200	14.42720000	0.00536100
H	-5.22941400	12.74886300	0.29615100
H	-9.13267800	-1.58857600	-0.19716800
H	-11.07153400	-3.13658500	-0.23400100
H	-6.48001300	-4.97068800	0.12663500
H	-12.70010800	-7.61764600	-0.04844600

H	-12.34249000	-10.09851300	0.13126800
H	-9.98193900	-10.96024100	0.32754000
H	-8.09362100	-9.33027200	0.33710900
H	1.87143100	-3.72117100	-0.48710700
H	7.98585400	-4.90275500	-0.43451500
H	10.33913600	-4.99789100	0.31182700
H	12.88144300	-4.45871100	0.36323700
H	14.50780100	-2.60929300	0.30303800
H	15.99073200	-1.68412200	1.46862900

WLK-5

N	1.98526900	0.26043100	-0.00706400
C	0.63068500	-0.14844700	-0.03155900
C	3.03098300	-0.70452000	-0.01897900
C	2.31677200	1.64702000	0.02659000
C	1.65920700	2.55897400	-0.80606600
C	1.98810900	3.90754500	-0.76837800
C	2.98883000	4.38940800	0.08729100
C	3.64604300	3.46424200	0.91014300
C	3.31362700	2.11578500	0.88826900
C	0.23742600	-1.28275800	-0.75664400
C	-1.09021300	-1.67919000	-0.77403000
C	-2.08174200	-0.96190300	-0.08419100

C	-1.68116300	0.18436700	0.62062000
C	-0.35148200	0.57771900	0.65566300
C	4.16989100	-0.50640700	-0.80775400
C	5.19755500	-1.44034900	-0.80590500
C	5.11883500	-2.60734000	-0.03374500
C	3.97155500	-2.79768900	0.74884400
C	2.94684800	-1.86115100	0.76543500
C	3.33642300	5.82886800	0.12191300
C	6.21135700	-3.60813800	-0.04488600
C	2.36859600	6.80663900	-0.04866800
C	2.70656900	8.16861000	-0.01705400
C	4.04029400	8.57229400	0.19533200
C	5.02168500	7.57663100	0.36909900
C	4.67644200	6.24508500	0.33120900
C	1.67309500	9.16373300	-0.19570300
N	2.13208900	10.51614900	-0.13817600
C	3.49114700	10.81981900	0.07567400
N	4.41257300	9.89928100	0.23627900
C	1.18103600	11.51893700	-0.30303800
C	1.53005100	12.82357600	-0.26413000
C	2.90111000	13.18103800	-0.04990700
C	3.83701100	12.21242900	0.11235500
O	0.46694000	8.93178700	-0.38849000
C	7.56311000	-3.19137800	-0.15577000
C	8.58518900	-4.11279900	-0.16763800
C	8.30140900	-5.48615200	-0.06695700
C	6.96464700	-5.91958800	0.04740500
C	5.93409300	-4.96340300	0.05370100

C	9.37694900	-6.44815800	-0.07853400
N	8.95629700	-7.81170600	0.02887800
C	7.59303800	-8.14964300	0.13529100
N	6.63204900	-7.25655600	0.14502000
C	9.94884400	-8.78647500	0.02248900
C	9.63868500	-10.09811400	0.11839100
C	8.26506900	-10.49092600	0.22826500
C	7.28853200	-9.54932100	0.23561600
O	10.58888900	-6.18588100	-0.17159600
C	-3.47688400	-1.43673400	-0.09572200
C	-3.78493700	-2.77060400	-0.19413600
C	-5.11664600	-3.27493900	-0.23557100
C	-6.23221100	-2.47626600	-0.17882800
C	-5.97684500	-1.06202100	-0.05790600
C	-4.61103200	-0.54230300	-0.01222200
N	-6.92839800	-0.15126400	0.01834300
S	-6.15594100	1.42076400	0.14965800
N	-4.52435900	0.77331100	0.09610200
C	-7.57806200	-3.03186600	-0.23015000
C	-7.91261000	-4.35055600	-0.44940700
C	-9.29745000	-4.61105100	-0.41961800
C	-10.07874500	-3.50205000	-0.17822800
S	-9.05368900	-2.03533000	0.01887400
C	-11.50648700	-3.53355300	-0.11896600
C	-12.40980200	-2.55057500	0.13772800
C	-12.04801600	-1.21144700	0.46038400
N	-11.76434500	-0.10916100	0.71887800
C	-13.86854100	-2.84734600	0.19467500

O	-14.66369400	-2.22213800	0.87619800
O	-14.30490900	-3.91684000	-0.56187500
H	0.89565400	2.20521600	-1.48815800
H	1.48262500	4.59342200	-1.43850000
H	4.40320800	3.80821700	1.60568200
H	3.81909300	1.42008600	1.54705500
H	0.97506800	-1.84049300	-1.31951600
H	-1.36845700	-2.53864600	-1.37329100
H	-2.41888200	0.77044000	1.14954200
H	-0.06758500	1.45450400	1.22357300
H	4.24095800	0.37879000	-1.42775700
H	6.05883100	-1.27694400	-1.44355900
H	3.89742400	-3.67418200	1.38231700
H	2.08003400	-2.01600900	1.39614800
H	1.32582600	6.54793800	-0.18799100
H	6.04397400	7.89833600	0.52076900
H	5.44887400	5.49242600	0.44142800
H	0.17972000	11.14571800	-0.45821900
H	0.77426900	13.58568000	-0.39445800
H	3.18282000	14.22645300	-0.01860300
H	4.88377000	12.42530300	0.27697100
H	7.79019800	-2.13343200	-0.20458700
H	9.62160300	-3.80707000	-0.24373100
H	4.91805500	-5.33164400	0.11291800
H	10.94819800	-8.38662200	-0.06424400
H	10.42649800	-10.83855600	0.11152900
H	8.01377100	-11.54177600	0.30513400
H	6.23796500	-9.78993900	0.31623100

H	-2.97976100	-3.49401400	-0.21637600
H	-5.23322800	-4.34920300	-0.30043500
H	-7.18074600	-5.12340900	-0.63653100
H	-9.72042300	-5.59629900	-0.57363500
H	-11.91397300	-4.52994300	-0.27856400
H	-13.70458400	-4.15547600	-1.29196500

WLK-6

N	1.94081100	0.11400500	-0.05249200
C	0.54636200	-0.08147800	-0.08395100
C	2.83538100	-0.99639400	-0.05788200
C	2.48880100	1.43217800	-0.00981900
C	2.02862900	2.42496100	-0.88084500
C	2.57247000	3.70198300	-0.83817900
C	3.59791700	4.02623900	0.06158700
C	4.05663300	3.01910800	0.92193300
C	3.50909600	1.74270500	0.89370500
C	-0.00963100	-1.20119600	-0.72970200
C	-1.37772700	-1.38740400	-0.75787100
C	-2.25604300	-0.46936900	-0.15126000
C	-1.69521500	0.64888200	0.48861200
C	-0.32297900	0.83789900	0.52383400
C	3.97131000	-0.98228500	-0.87355300
C	4.85646800	-2.05243900	-0.86274000
C	4.63081300	-3.17357100	-0.05261600

C	3.48728600	-3.17754300	0.75814700
C	2.60589300	-2.10467500	0.76497300
C	4.17460100	5.39012800	0.10185600
C	5.57057500	-4.31950100	-0.05317500
C	3.38818600	6.50948700	-0.12196700
C	3.94216100	7.79859900	-0.08451000
C	5.31280600	7.98290300	0.18797600
C	6.10955700	6.84346100	0.41566000
C	5.55364800	5.58547900	0.37157600
C	3.09429000	8.94571900	-0.31980200
N	3.76474900	10.20606100	-0.25067600
C	5.14386800	10.28769100	0.02543100
N	5.89437600	9.23191000	0.23661800
C	2.99878900	11.34767000	-0.46825600
C	3.55410300	12.57850900	-0.42252200
C	4.95380200	12.71119800	-0.14580900
C	5.71052000	11.60570200	0.06817600
O	1.87653500	8.90997500	-0.56815200
C	6.96494900	-4.10164600	-0.19884400
C	7.84584400	-5.15896100	-0.20101600
C	7.37248600	-6.47484300	-0.05583800
C	5.99057600	-6.71097900	0.09338000
C	5.10592200	-5.61816200	0.08933100
C	8.30058800	-7.58005400	-0.05744100
N	7.69351300	-8.86650000	0.09680000
C	6.29864400	-9.00439500	0.23593200
N	5.47421300	-7.98377200	0.23558100
C	8.53767400	-9.97234700	0.10237200

C	8.04678200	-11.22347800	0.24192400
C	6.63397300	-11.41397400	0.38629700
C	5.80098600	-10.34324800	0.38237300
O	9.53521800	-7.49488700	-0.17847100
C	-3.69430200	-0.72812000	-0.22133600
N	-4.09363000	-1.82798400	-0.83703600
C	-5.41104300	-2.17210200	-0.96821200
C	-6.48020700	-1.44491900	-0.50230000
C	-6.12476300	-0.23511900	0.18918900
C	-4.72252200	0.13825900	0.33855300
N	-6.99315500	0.60104700	0.72657800
S	-6.08369700	1.91565000	1.45551600
N	-4.51786900	1.26722500	0.99241900
C	-7.85231200	-1.88205300	-0.69734200
C	-8.27361200	-2.97336800	-1.42545700
C	-9.66983600	-3.16716800	-1.42085900
C	-10.37206500	-2.23222100	-0.69173500
S	-9.25194800	-1.02621200	0.03761900
C	-11.79543200	-2.22931700	-0.56245800
C	-12.62510900	-1.40801400	0.13408600
C	-12.16783100	-0.34083000	0.95890300
N	-11.80459500	0.54109700	1.63167600
C	-14.09611600	-1.64278100	0.15395900
O	-14.82239800	-1.32599000	1.08119900
O	-14.62846300	-2.28220600	-0.94776800
H	1.25031500	2.18862600	-1.59636500
H	2.21906100	4.45097300	-1.53748400
H	4.82790400	3.24532300	1.64938200

H	3.86353300	0.98217700	1.57895800
H	0.64083400	-1.91558000	-1.21723000
H	-1.80272400	-2.24448400	-1.26285700
H	-2.34060800	1.36814500	0.97178700
H	0.08340400	1.70079000	1.03499600
H	4.15167800	-0.13269500	-1.52078400
H	5.71753100	-2.03144300	-1.52080900
H	3.30491300	-4.01670800	1.41944600
H	1.74009200	-2.11664400	1.41579300
H	2.32463100	6.42198000	-0.30878100
H	7.16263000	6.99678500	0.61331400
H	6.18697000	4.71893600	0.52417000
H	1.95814400	11.14017800	-0.66821200
H	2.93976200	13.45144400	-0.59444600
H	5.40074800	13.69714100	-0.10889200
H	6.76931700	11.64781300	0.28096500
H	7.33837500	-3.08832200	-0.28236000
H	8.91331100	-5.00582500	-0.30332900
H	4.04943100	-5.83664800	0.17636200
H	9.58158600	-9.72119500	-0.01277900
H	8.72143000	-12.06834600	0.24359800
H	6.23800000	-12.41597500	0.49817900
H	4.72882400	-10.42975200	0.48759400
H	-5.56851000	-3.10846700	-1.48671900
H	-7.59770700	-3.62437500	-1.96140300
H	-10.15839400	-3.97958300	-1.94481300
H	-12.26950600	-3.05115000	-1.09542400
H	-14.07455500	-2.21972700	-1.74754600

References:

1. Y. Gao, S. Dai, J. Zhu, L. Wu, L. Han, Y. Li, Q. Ye and Y. Cui, *J. Photochem. Photobiol., A*, 2023, **438**, 114563.
